# Human HMGN1 and HMGN2 are not required for transcription-coupled DNA repair

**DOI:** 10.1038/s41598-020-61243-4

**Published:** 2020-03-09

**Authors:** Katja Apelt, Iris Zoutendijk, Dennis Y. Gout, Annelotte P. Wondergem, Diana van den Heuvel, Martijn S. Luijsterburg

**Affiliations:** Department of Human Genetics, Leiden University Medical Center, Einthovenweg 20, 2333 ZC Leiden, The Netherlands

**Keywords:** Nucleotide excision repair, Transcription

## Abstract

Transcription-coupled repair (TCR) removes DNA lesions from the transcribed strand of active genes. Stalling of RNA polymerase II (RNAPII) at DNA lesions initiates TCR through the recruitment of the CSB and CSA proteins. The full repertoire of proteins required for human TCR – particularly in a chromatin context - remains to be determined. Studies in mice have revealed that the nucleosome-binding protein HMGN1 is required to enhance the repair of UV-induced lesions in transcribed genes. However, whether HMGN1 is required for human TCR remains unaddressed. Here, we show that knockout or knockdown of HMGN1, either alone or in combination with HMGN2, does not render human cells sensitive to UV light or Illudin S-induced transcription-blocking DNA lesions. Moreover, transcription restart after UV irradiation was not impaired in HMGN-deficient cells. In contrast, TCR-deficient cells were highly sensitive to DNA damage and failed to restart transcription. Furthermore, GFP-tagged HMGN1 was not recruited to sites of UV-induced DNA damage under conditions where GFP-CSB readily accumulated. In line with this, HMGN1 did not associate with the TCR complex, nor did TCR proteins require HMGN1 to associate with DNA damage-stalled RNAPII. Together, our findings suggest that HMGN1 and HMGN2 are not required for human TCR.

## Introduction

Nucleotide excision repair (NER) is a versatile DNA repair system that removes a wide range of helix-distorting DNA lesions from our genome. The NER pathway is initiated by two different damage-recognition mechanisms. While transcription-coupled repair (TCR) preferentially removes DNA lesions from actively transcribed DNA strands^1^, global genome repair (GGR) removes helix-distorting lesions such as cyclobutane pyrimidine dimers (CPDs) throughout the genome^2^. A wide-range of DNA lesions is recognized and repaired by NER, including ultra-violet (UV) light-induced photolesions, and transcription-blocking DNA lesions induced by natural compounds, such as Illudin S^3–5^.

TCR is initiated by the stalling of elongating RNA polymerase II (RNAPIIo) at DNA lesions, which subsequently triggers the recruitment of the Cockayne syndrome proteins CSB and CSA, as well as UVSSA^6,7^. GGR, on the other hand, involves the XPC and DDB2 damage-recognition proteins that constantly probe genomic DNA for the presence of lesions^8^. Following these initials steps, the TCR and GGR pathways funnel into a common molecular mechanism that involves the assembly of the pre-incision complex, including the TFIIH complex, XPA, RPA, and the endonucleases XPG and ERCC1-XPF, ultimately leading to the incision of the lesion^9^.

Genomic DNA is packaged by histone and non-histone proteins into chromatin, which limits the accessibility of repair proteins to genomic DNA and therefore complicates efficient DNA repair^10^. Emerging evidence suggests that modulating chromatin structure is a key step in mounting an efficient cellular response to DNA lesions. UV-dependent chromatin changes during GGR are facilitated by ATP-dependent chromatin-remodeling complexes, including INO80^11^, ALC1^12^ and CHD1^13^. Several studies have also implicated a role for chromatin-modifying activities associated with TCR-dependent transcription restart in human cells, including histone chaperones FACT and HIRA, and chromatin remodeling factor SNF2H^14–18^. Furthermore, the CSB protein is a DNA-dependent ATPase with the ability to remodel nucleosomes *in vitro*^19^, which could contribute to TCR *in vivo*^20^.

The high-mobility group N (HMGN) family members are architectural proteins that modulate chromatin structure. The HMGN family consists of five members of which HMGN1 and HMGN2 are the most abundant and best studied proteins^21^. All HMGN proteins contain a nucleosome-binding domain (NBD) and a C-terminal chromatin-unfolding domain (CHUD)^22,23^. The HMGN proteins associate with nucleosomes and destabilize higher-order chromatin structure by modulating the binding of linker histone H1, thereby increasing the accessibility of nucleosomal DNA^24,25^.

Studies in mouse embryonic fibroblasts have revealed that HMGN1 has the ability to increase the cellular transcription potential by unfolding higher-order chromatin structure^26^. Interestingly, mouse embryonic fibroblasts deficient in HMGN1 show a decreased repair rate of UV-induced DNA lesions particularly in transcribed genes^27^, suggesting an involvement in modulating chromatin structure during murine TCR. Rather than a specific TCR factor, murine HMGN1 seems to have a more general role in enabling DNA repair in chromatin, since mouse embryonic fibroblasts deficient in HMGN1 show not only defects in the repair of UV-induced lesions^27^, but also in the repair of alkylated DNA bases induced by MMS^28^, and DNA double-strand breaks^29^.

Most studies addressing the versatile roles of HMGN1 have focused on mouse cells, while our current understanding of the function of HMGN1 in human cells is fairly limited. Although often assumed^9,30^, experimental evidence showing that HMGN1 has a role in human TCR – similar to its murine counterpart - is lacking. In this study, we established knockout cells for HMGN1 alone or in combination with HMGN2 in two independent human cell-lines. Functional analysis revealed that, in contrast to mouse cells, human HMGN1 and HMGN2 are dispensable for human TCR.

## Results

### Generation of human HMGN1 knockout cells

**Figure 1:**
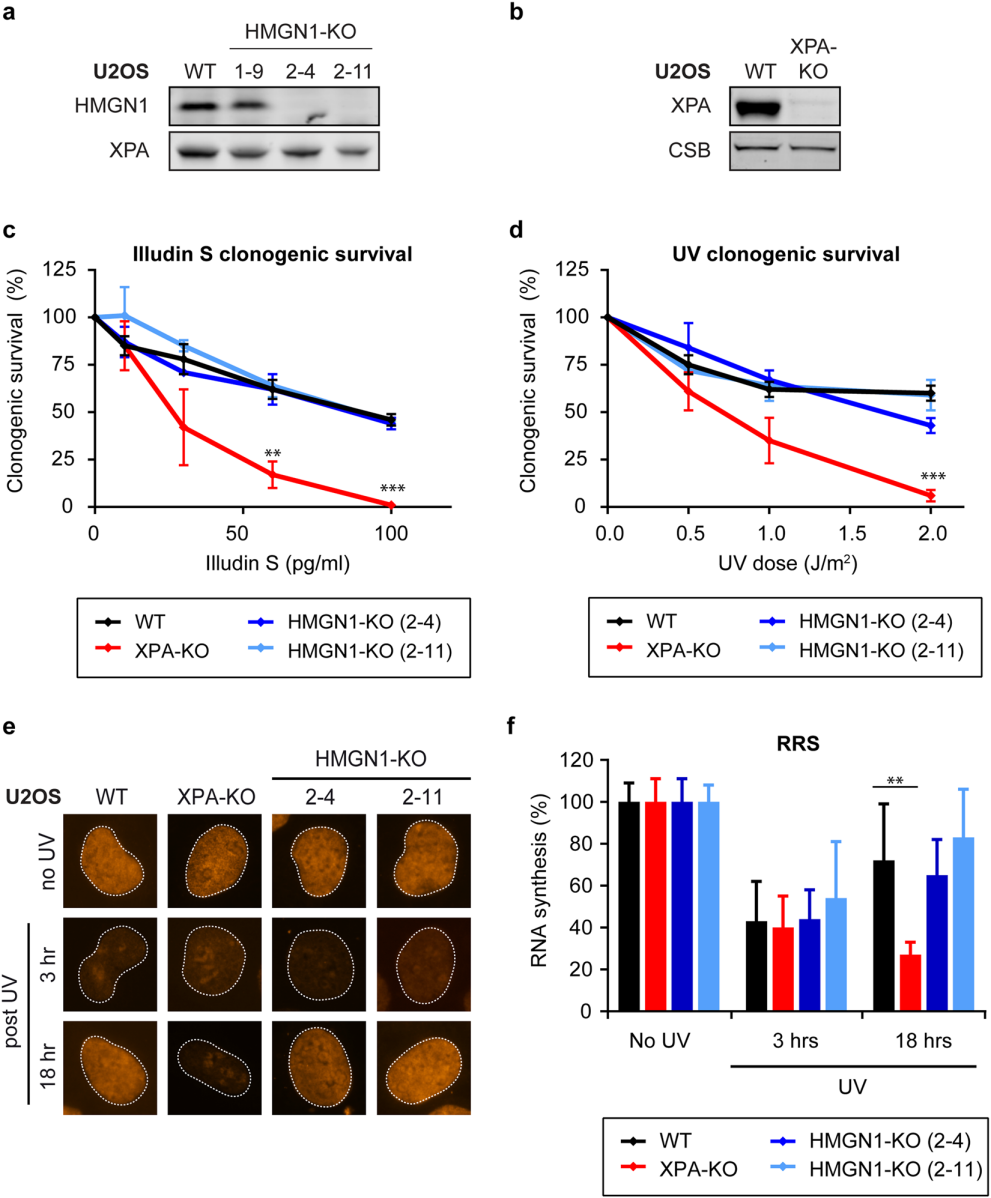
HMGN1 knockout does not impact human TCR. (**a**) Western blot analysis of U2OS WT and HMGN-KO clones or (**b**) U2OS WT and XPA-KO clone. (**c**) Clonogenic Illudin S survival or (**d**) clonogenic UV survival of WT, XPA-KO, and HMGN1-KO cell lines. Data represent mean ± SEM of three independent experiments. (**e**) Representative microscopy images, and (**f**) Quantification of RRS after UV on the WT, XPA-KO, and HMGN1-KO cell lines. Data represent mean ± SEM of three independent experiments. Uncropped Western blot data is shown in the Supplementary Information File.

Studies in mouse embryonic fibroblasts have revealed a role of HMGN1 in enhancing the repair rate of UV-induced DNA lesions in particular from transcribed genes^27^, suggesting a possible involvement of HMGN1 in murine transcription-coupled repair (TCR). However, whether HMGN1 is involved in human TCR has remained unexplored. To study a potential role of HMGN1 in human cells, we used CRISPR/Cas9-mediated genome editing to generate HMGN1 knock-out (KO) cells. To this end, we transfected U2OS cells with vectors encoding HMGN1-specific sgRNAs and Cas9 after which clones were isolated and screened (Fig. 1a). Western blot analysis using HMGN1-specific antibodies confirmed the knock-out of HMGN1 in two independent clones (Fig. 1a; clone 2–4 and 2–11). These findings reveal that loss of HMGN1 is viable in human cells and provide a new tool to study the role of HMGN1 in human cells.

### Human HMGN1-KO cells are resistant to Illudin S and UV

Elongating RNA polymerase II (RNAPIIo) molecules are unable to efficiently bypass DNA lesions that block transcription, including those inflicted by the sesquiterpene drug Illudin S^4^ or by ultra-violet (UV) light, which inflicts photoproducts such as CPDs^3^. To overcome the obstacle posed by these DNA lesions, human cells fully depend on TCR to remove these lesions during transcription. In particular Illudin S-induced lesions are removed exclusively by TCR^4^. To directly compare our HMGN1-KO cells in the same genetic background with TCR-deficient cells, we used CRISPR/Cas9-mediated genome editing to generate XPA knockout (KO) cells. Western blot analysis confirmed the knockout of XPA (Fig. 1b). Moreover, clonogenic survivals assays in which cells were exposed to increasing concentrations of Illudin S (Fig. 1c), or increasing doses of UV-C light (Fig. 1d), confirmed that XPA-KO cells were highly sensitive to these DNA-damaging agents compared to parental wild-type (WT) cells. In contrast, two independent HMGN1-KO clones, which were included in parallel, showed no sensitivity to either Illudin S or UV irradiation compared to WT cells (Fig. 1c,d). These findings show that, in contrast to murine cells^27^, loss of HMGN1 in human osteosarcoma cells does not cause sensitivity to transcription-blocking DNA damage.

### Human HMGN1-KO cells show normal transcription recovery after UV

In addition to sensitivity to transcription-blocking DNA damage, another hallmark of TCR-deficient cells is their inability to restart transcription after UV irradiation^31^. To quantify the ability of our HMGN1-KO cells to restart transcription, we performed recovery of RNA synthesis (RRS) experiments. To this end, we either mock treated or exposed cells to UV-C light (6 J/m2). Nascent transcripts were pulse-labeled for 1 hour with the cell-permeable thymine analogue 5-ethynyl uridine (5-EU). Nascent transcripts containing 5-EU were visualized via copper-catalyzed click chemistry of an azide-coupled fluorescent dye. Microscopic analysis revealed that WT cells showed a pronounced inhibition of transcription at 3 hours after UV, due to stalling of RNAPII, while significant transcription restart could be detected at 18 hours after UV irradiation (Fig. 1e,f). This transcription restart was completely blocked in TCR-deficient XPA-KO cells due to their inability to clear transcription-blocking UV-induced lesions from the genome (Fig. 1e,f). In contrast, two independent HMGN1-KO clones showed a normal restart of transcription after UV irradiation, suggesting that these cells are not deficient in TCR.

### HMGN2 does not compensate for HMGN1 in TCR

**Figure 2:**
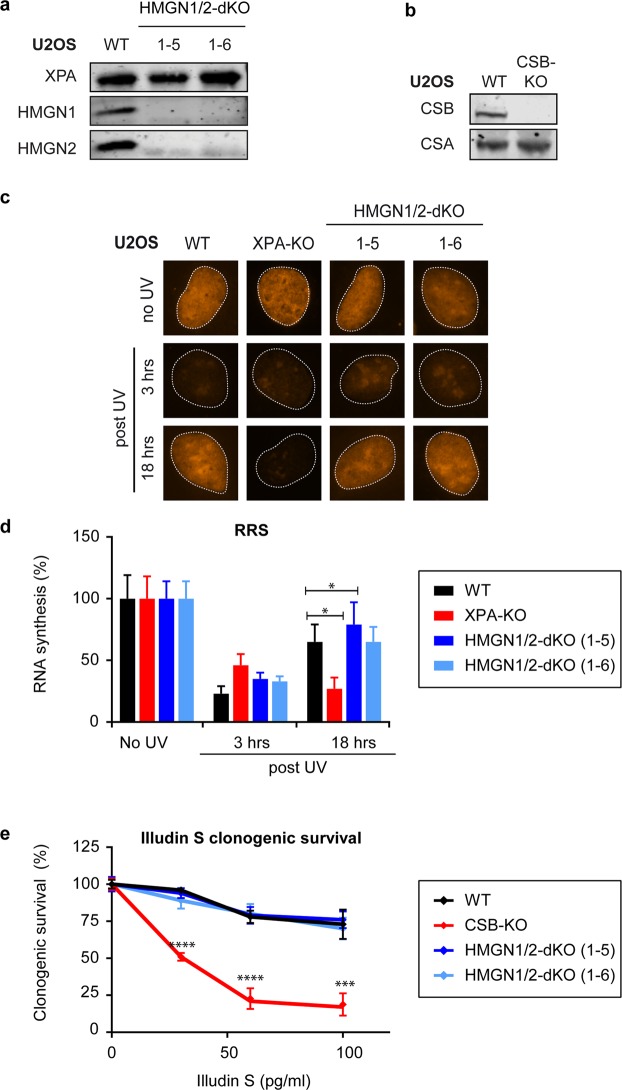
HMGN1 and HMGN2 double knockout does not impact human TCR in U2OS cells. (**a**) Western blot analysis of U2OS WT and HMGN1/HMGN2-dKO clones or (**b**) U2OS WT and CSB-KO clone. (**c**) Representative microscopy images, and (**d**) Quantification of RRS after UV on the WT, XPA-KO, and HMGN1/HMGN2-dKO cell lines. Data represent mean ± SEM of five independent experiments. (**e**) Clonogenic Illudin S survival of U2OS WT, CSB-KO, and HMGN1/HMGN2-dKO cell lines. Data represent mean ± SEM of five independent experiments. Uncropped Western blot data is shown in the Supplementary Information File.

Although HMGN1-deficient mouse embryonic fibroblasts are sensitive to UV irradiation^27^, we did not observe this phenotype in human HMGN1-KO cells. It has been reported that HMGN2 can functionally compensate for HMGN1 in murine cells^32^, and we therefore considered that a similar functional redundancy may mask the role of HMGN1 in human TCR. To test this possibility, we generated HMGN1/HMGN2 double knock-out (dKO) cells by CRISPR-Cas9-mediated genome editing. To this end, U2OS cells were co-transfected with vectors encoding sgRNAs targeting both *HMGN1* and *HMGN2* genes, as well as with a vector encoding the Cas9 protein. Cells were sorted by flow cytometry based on GFP expression encoded on the Cas9 vector, and clones were isolated and screened. Western blot analysis using antibodies specific for human HMGN1 and HMGN2 confirmed the loss of both HMGN proteins in our selected KO clones (Fig. 2a; clones 1–5 and 1–6). In addition, TCR deficient CSB-KO cells were generated in parallel (Fig. 2b). Importantly, two independent HMGN1/HMGN2-dKO clones showed a normal transcription restart after UV irradiation in RRS experiments, while XPA-KO cells, included in parallel failed to resume transcription (Fig. 2c,d). Furthermore, both HMGN1/HMGN2-dKO clones were resistant to Illudin S-induced DNA lesions, while CSB-KO cells, which were included as a control, were highly sensitive to transcription-blocking lesions induced by this compound (Fig. 2e).

**Figure 3:**
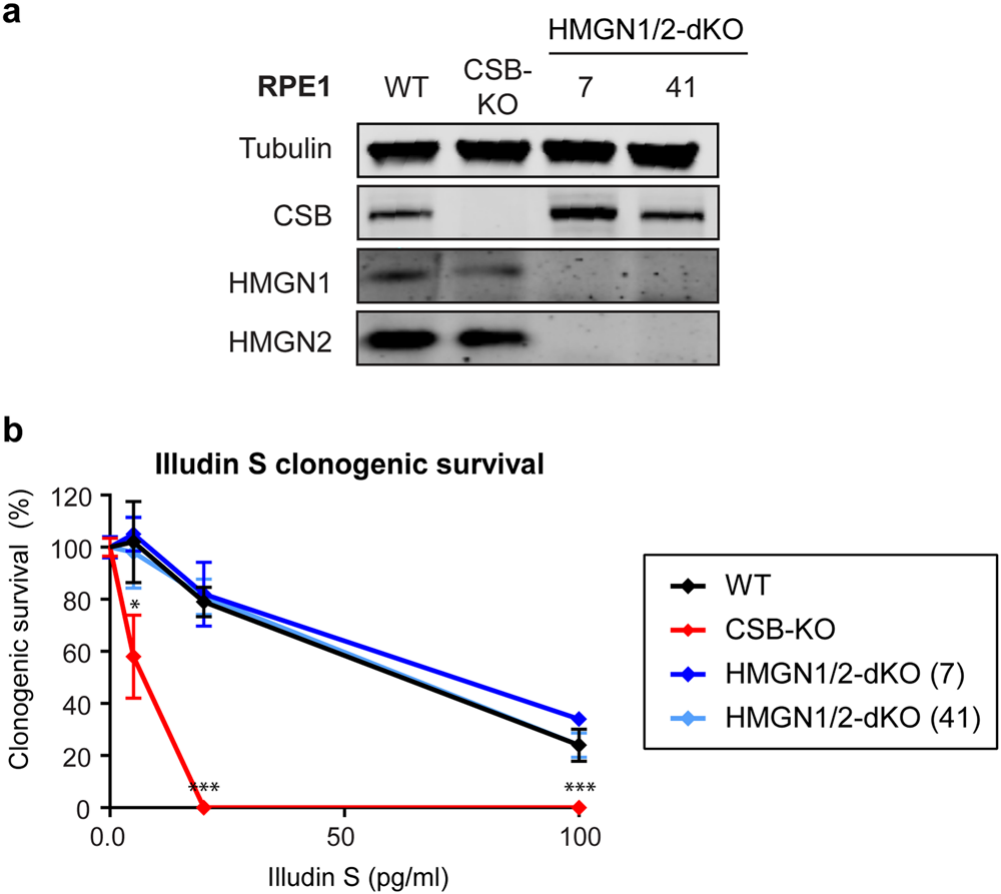
Knockout of HMGN1 and HMGN2 does not cause Illudin S sensitivity in RPE1-hTERT cells. (**a**) Western blot analysis of RPE1-hTERT WT, CSB-KO clone and two HMGN1/HMGN2-dKO clones. (**b**) Clonogenic Illudin S survival on RPE1-hTERT WT, CSB-KO, and two HMGN1/HMGN2-dKO clones.

To confirm that this lack of phenotype is not specific to osteosarcoma cells, we generated additional HMGN1/HMGN2-dKO cells in hTERT-immortalized human retinal pigment epithelial cells (RPE1) by CRISPR-Cas9-mediated genome editing. Western blot analysis confirmed the loss of HMGN1 and HMGN2 expression in two selected dKO clones (Fig. 3a; clone 7 and 41). As a control, we also generated CSB-KO cells in RPE1-hTERT cells and confirmed loss of expression using CSB-specific antibodies (Fig. 3a). In line with results in U2OS, the two independent HMGN1/HMGN2-dKO clones in RPE1-hTERT cells were not sensitive to Illudin S compared to wild-type RPE1-hTERT cells even when cells were exposed to high concentrations (100 pg/mL) of Illudin S that resulted in ~70% cell death in WT cells. In contrast, CSB-KO cells showed a dose-dependent increase in Illudin S sensitivity under similar condition and did not survive beyond 25 pg/mL (Fig. 3b).

These findings suggest that HMGN2 does not functionally compensate for HMGN1, and that neither HMGN protein is required for TCR in human cells.

### Knockdown of HMGN1 or HMGN2 does not cause TCR defects in human cells

**Figure 4:**
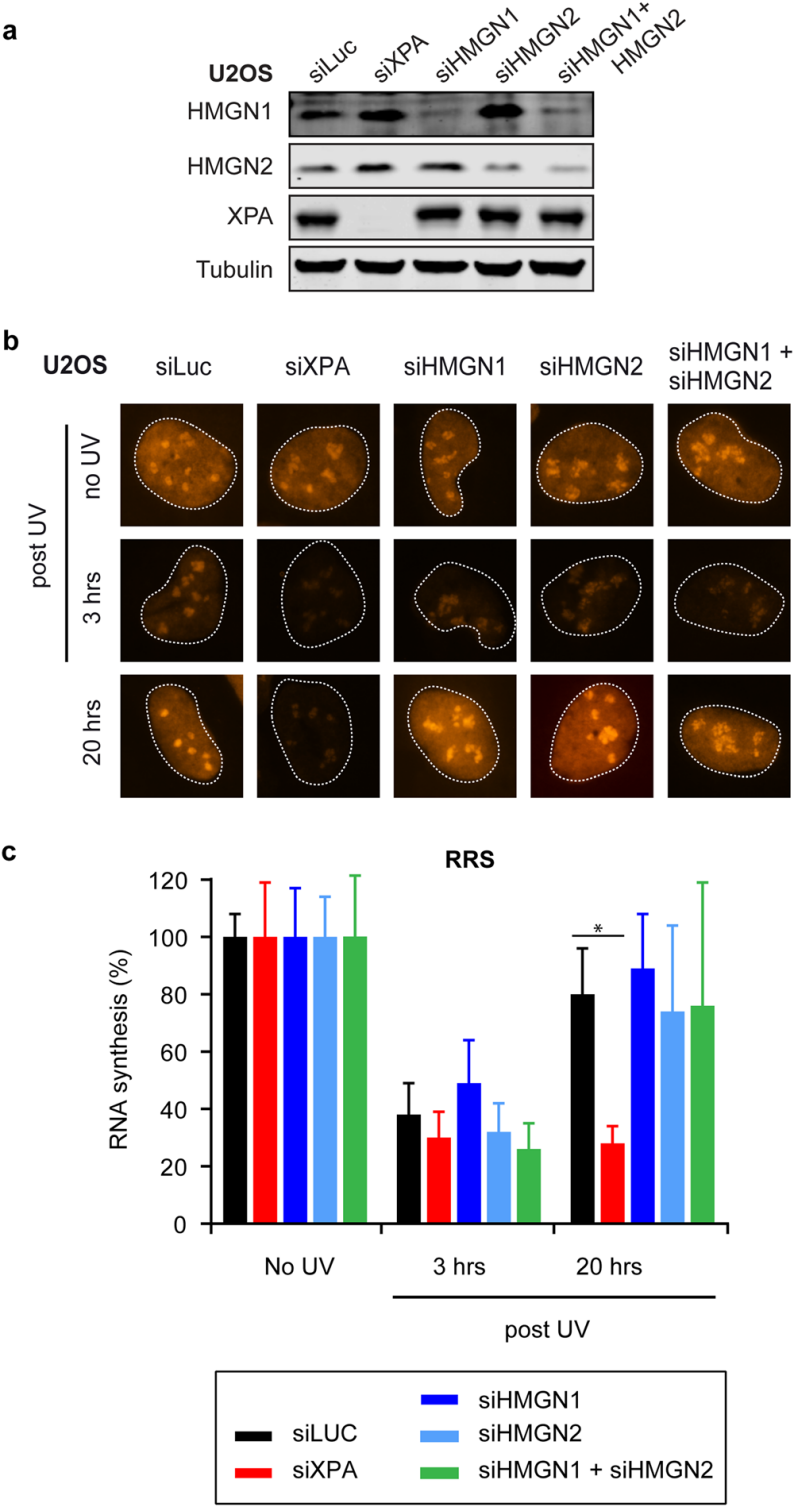
HMGN1 and HMGN2 knockdown does not impact human TCR. (**a**) Western blot analysis of U2OS cells transfected with the indicated siRNAs. (**b**) Representative microscopy images, and (**c**) Quantification of RRS after UV on U2OS cells transfected with the indicated siRNAs. Data represent mean ± SEM of three independent experiments. Uncropped Western blot data is shown in the Supplementary Information File.

Our previous findings using independently generated HMGN1-KO clones, or HMGN1/HMGN2-dKO clones in two different cell types revealed no signs of TCR deficiency (Figs. 1 and 2). To rule out the possibility that these KO cells genetically adapted during their clonal expansion, we decided to employ a more acute way of removing the expression of HMGN proteins. To this end, we employed specific siRNAs to knockdown the expression of the HMGN proteins or XPA as a control. Western blot analysis showed that we achieved efficient knockdown of HMGN1, HMGN2, or XPA within a time-course of four days (Fig. 4a). However, as observed in our HMGN-KO cells, the knockdown of either HMGN1, HMGN2, or the combined knockdown of both HMGN proteins did not affect the restart of transcription after UV irradiation in RRS experiments, while knockdown of XPA fully impaired this process (Fig. 4b,c). These findings suggest that acute knockdown of HMGN proteins, like genetic knockout of HMGN proteins, does not cause a deficiency in human TCR.

### Knockout of HMGN1 and HMGN2 does not delay TCR in human cells

In the previous experiments, we found that knockout of HMGN1 and HMGN2 did not affect TCR by measuring endpoints such as transcription recovery at 18 hours after UV irradiation. However, it cannot be excluded that HMGN proteins modulate the kinetics of TCR rather than the absolute activity measured at such endpoints.

**Figure 5:**
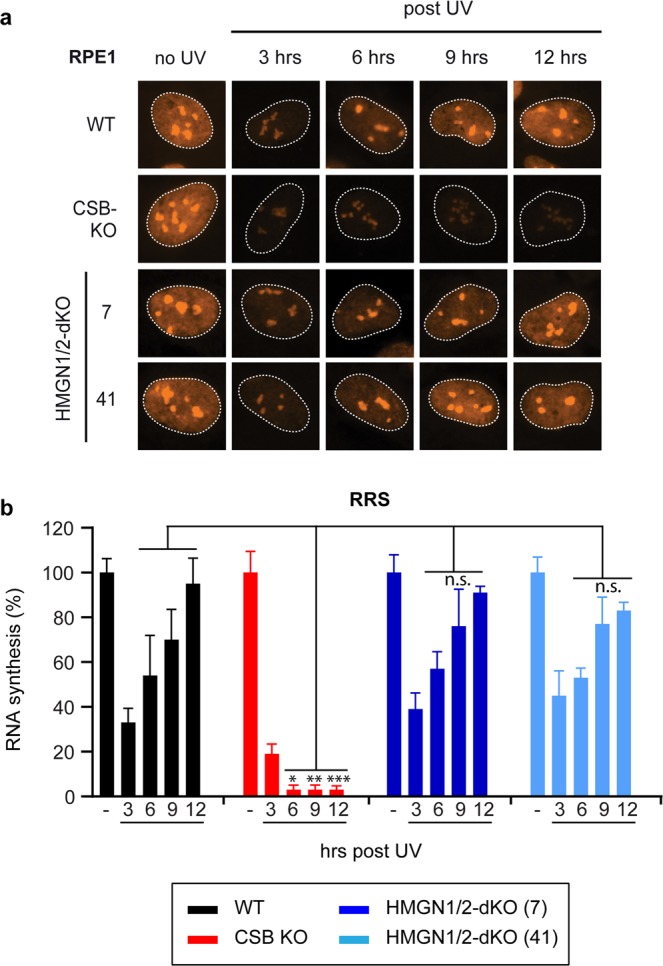
Knockout of HMGN1 and HMGN2 does not delay TCR in human fibroblasts. (**a**) Representative microscopy images, and (**b**) Quantification of RRS after UV on the RPE1-hTERT WT, CSB-KO, and HMGN1/HMGN2-dKO cell lines. Data represent mean ± SEM of two independent experiments. Uncropped western blot data is shown in the Supplementary Information File.

To measure TCR kinetics, we measured the rate of transcription restart by labelling nascent RNA at different time-points after UV-C irradiation in RPE1-hTERT cells. Microscopic analysis revealed a strong inhibition of transcription at 3 hours after UV-C in all cell types (Fig. 5a,b). A gradual increase in nascent transcription occurred over the course of several hours with a near complete recovery of transcription around 12 hours after UV-C in wild-type cells (Fig. 5a,b). Within the same time-course, we did not detect any restart of transcription in CSB-KO cells as expected. However, the kinetics of transcription restart in the HMGN1/HMGN2-dKO after UV-C were very similar to WT cells at all time-points analyzed (Fig. 5a,b). These findings show that loss of HMGN1 and HMGN2 does not affect the kinetics of TCR in human epithelial cells.

### Knockout of HMGN1 and HMGN2 does not affect UV sensitivity

**Figure 6:**
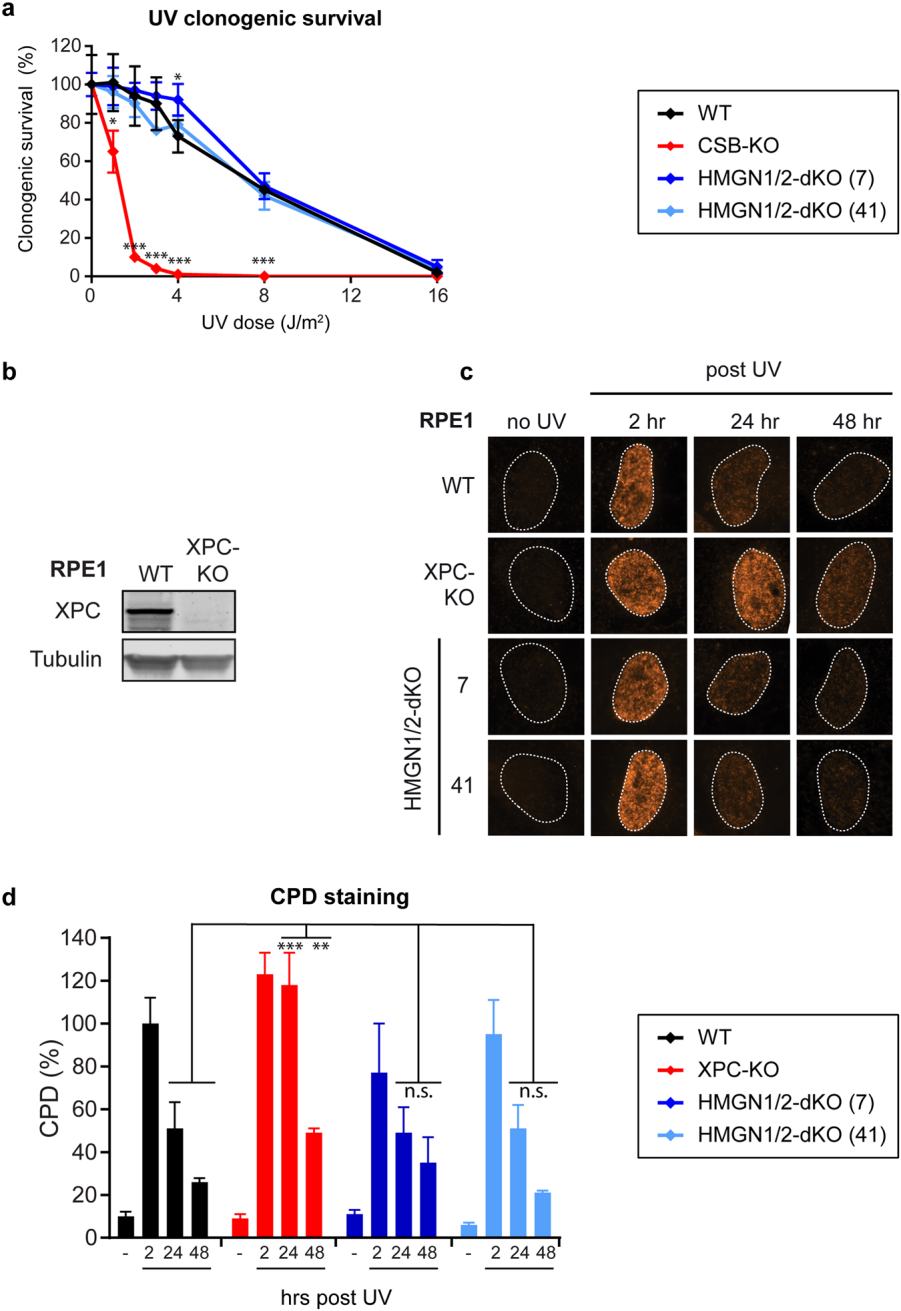
Knockout of HMGN1 and HMGN2 does not affect UV sensitivity (**a**) Clonogenic UV survival of RPE1-hTERT WT, CSB-KO, and two HMGN1/HMGN2-dKO clones. (**b**) Western blot analysis of RPE1-hTERT WT and XPC-KO clone (**c**) Representative microscopy images, and (**d**) Quantification of CPD staining after UV on the RPE1-hTERT WT, XPC-KO and two HMGN1/HMGN2-dKO clones. Data represent mean ± SEM of two independent experiments. Uncropped western blot data is shown in the Supplementary Information File.

Previous findings showed that HMGN1-deficient mouse embryonic fibroblasts are sensitive to UV irradiation (measured up to 16 J/m2) in cell proliferation assays^27^. To measure UV sensitivity, we performed clonogenic survival assays in RPE1-hTERT cells exposed to increasing doses of UV-C ranging up to 16 J/m2. Both WT and HMGN1/HMGN2-dKO cells displayed highly comparable survival at all UV doses analyzed (1, 2, 3, 4, 8, 16 J/m2), which decreased in a dose-dependent manner until none of the cells survived at the highest dose tested even in WT cells (Fig. 6a). In contrast, CSB-KO cells showed less than 10% survival after irradiation with 2 J/m2 (Fig. 6a). These findings demonstrate that loss of HMGN1 and HMGN2 does not affect the sensitivity to UV irradiation even under conditions that provide a large dynamic window to detect a more subtle phenotype.

The lack of UV-sensitive phenotype suggests that HMGN1/HMGN2-dKO are also not impaired in the repair of UV-induced DNA lesions by GGR. To address this more directly, we measured the removal of CPDs, which is the most abundant DNA lesion generated by UV-C irradiation. As a control, we generated GGR-deficient XPC-KO cells, which were confirmed by western blot analysis (Fig. 6b). We applied immunofluorescent labeling using antibodies against CPDs at different timepoints after UV irradiation to detect DNA lesions in genomic DNA in intact RPE1-hTERT cells. Both WT cells and HMGN1/HMGN2-dKO repaired the majority of the CPDs in the genome within the first 48 hours after UV (Fig. 6c,d). In contrast, XPC-KO cells failed to clear CPDs from their genome within the first 24 hours after UV in line with their GGR deficiency. Notably, we detected a two-fold reduction in CPD signal at 48 hours in XPC-KO cells, which is likely due to a dilution of the DNA damage load during cell division. In summary, loss of HMGN1 and HMGN2 does not affect the cellular response to UV-induced DNA lesions.

### Human HMGN1 does not associate with stalled RNAPII and TCR proteins

**Figure 7:**
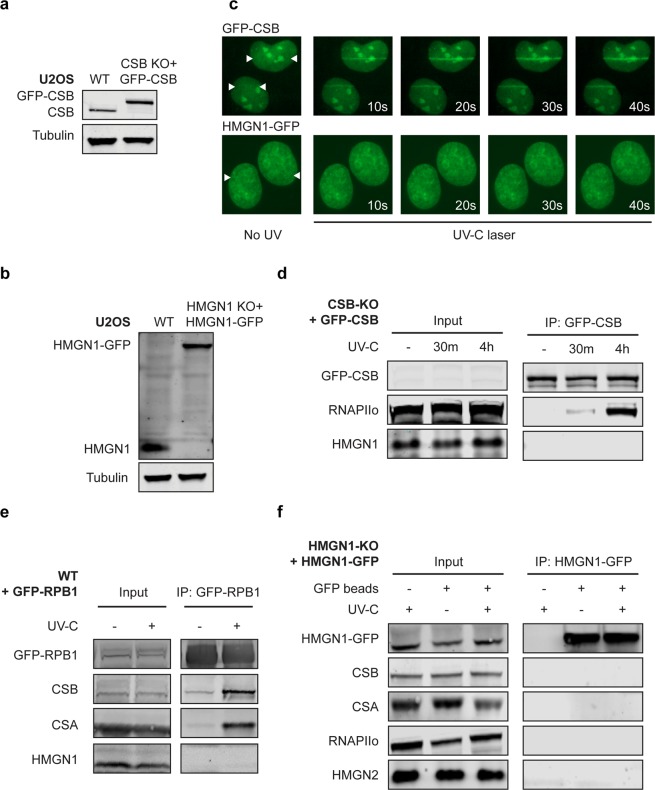
HMGN1 does not associate with the TCR complex. (**a**) Western blot analysis of U2OS WT and CSB-KO + GFP-CSB or (**b**) U2OS WT and HMGN1-KO + HMGN1-GFP. (**c**) Live-cell imaging on GFP-CSB or HMGN1-GFP after induction of UV-C-laser-induced DNA damage. The position of the laser track is indicated by white arrows. (**d**) Co-IP of GFP-CSB in unirradiated or UV-irradiated cells at the indicated time-points. (**e**) Co-IP of GFP-RPB1 in unirradiated or UV-irradiated cells. (**f**) Co-IP of HMGN1-GFP in unirradiated or UV-irradiated cells at the indicated time-points. Uncropped Western blot data is shown in the Supplementary Information File.

The stalling of RNAPII at DNA lesions triggers the association of TCR proteins, including CSB, to initiate repair. We decided to monitor the possible association of HMGN1 with TCR proteins using two independent approaches. We first employed irradiation of cells with a pulsed 266 nm UV-C laser on a live-cell imaging set-up in which all glass optics were replaced by quartz optics to allow full UV-C transmission^33^. To monitor recruitment of proteins using this set-up, we stably expressed either GFP-CSB or HMGN1-GFP at endogenous levels in the corresponding KO clones (Fig. 7a,b). While UV-C laser-induced DNA damage readily triggered recruitment of GFP-CSB into locally irradiated regions, we failed to detect recruitment of HMGN1-GFP to sites of UV-C laser-induced DNA damage (Fig. 7c). Importantly, GFP-tagged human HMGN1 was nuclear and showed a clear chromatin-bound pattern (Fig. 7c). Additionally, the same GFP-tagged HMGN1 cDNA was previously shown to complement the phenotype of murine HMGN1-deficient cells demonstrating its functionality^27^.

**Figure 8:**
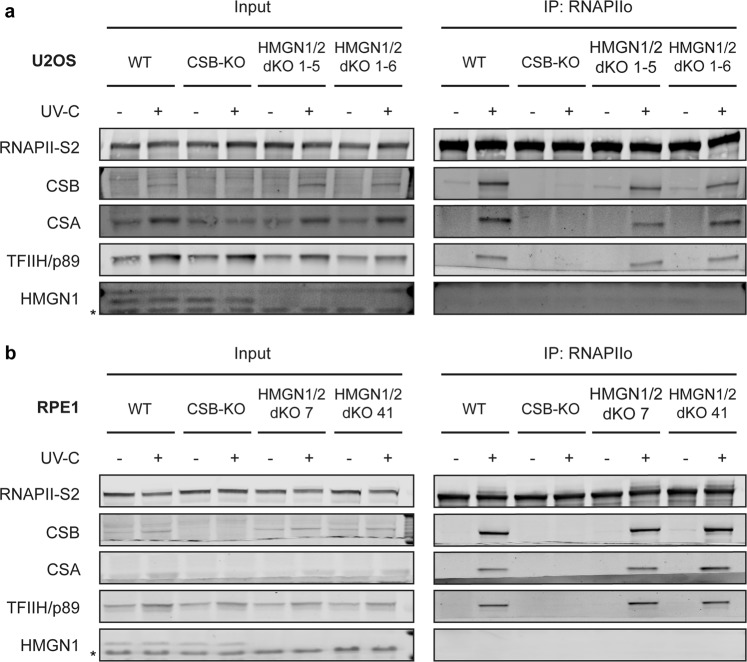
HMGN1 and HMGN2 are not required for TCR complex assembly. (**a**) Co-IP of endogenous RNAPIIo in unirradiated or UV-irradiated U2OS WT, CSB-KO and HMGN1/HMGN2-dKO cells or (**b**) RPE1-hTERT WT, CSB-KO and HMGN1/HMGN2-dKO cells. Uncropped Western blot data and Co-IP repeats are shown in the Supplementary Information File.

As an alternative approach, we employed immunoprecipitation under native conditions after UV irradiation using the same cell lines that were used for live-cell imaging. While we could clearly detect a UV-induced association of RNAPIIo after immunoprecipitation of GFP-CSB, we failed to detect the association of endogenous HMGN1 under the same conditions (Fig. 7d). Similarly, immunoprecipitation of GFP-tagged RNAPII from cells stably expressing GFP-RPB1^34^, revealed robust UV-induced interactions with both CSB and CSA (Fig. 7e), demonstrating that our conditions do allow us to detect interactions with TCR proteins after UV irradiation. However, we could not detect an interaction between GFP-RPB1 and endogenous HMGN1 under these conditions (Fig. 7e). Reciprocal immunoprecipitation experiments on HMGN1-GFP also did not show any interactions with CSB, CSA, RNAPIIo, or HMGN2 in unirradiated or UV-irradiated cells (Fig. 7f). Finally, we immunoprecipitated endogenous RNAPIIo, which strongly interacted with CSB, CSA and the TFIIH complex after UV irradiation (Fig. 8a). However, while all these interactions with RNAPIIo were abolished in CSB-KO cells (Fig. 8a), in line with the essential role of this protein in TCR, the UV-induced association of these TCR proteins with RNAPIIo was not affected in two independent HMGN1/HMGN2-dKO clones in either osteosarcoma (Fig. 8a, Supplementary Fig. 2A,B) or retinal epithelial cells (Fig. 8b, Supplementary Fig. 3A,B). These findings show that human HMGN1 does not interact with DNA damage-stalled RNAPII and associated TCR proteins, and that both HMGN1 and HMGN2 are dispensable for human TCR.

## Discussion

Although often inferred, based on studies in mouse embryonic fibroblasts^27^, it is currenly unknown if the nucleosome-binding protein HMGN1 has a role in modulating chromatin structure to enhance transcription-coupled DNA repair (TCR) in human cells. In the current study, we generated human HMGN1 knockout (KO) cells to directly adress this unanswered question. Functional analysis of human HMGN1-KO cells revealed that this nucleosome-binding protein is dispensible for human TCR. Our findings suggest that the role of murine HMGN1 in TCR is not functionally conserved in human cells.

### Human HMGN1 and HMGN2 are not involved in TCR

Several key reviews on TCR mention HMGN1 and list this nucleosome-binding protein as a key factor that modulates human TCR^9,30,35^. However, it should be emphasized that while HMGN1-deficient mouse embryonic fibroblasts show decreased repair of UV-induced DNA lesions from active genes^27^, a functional role of HMGN1 in human TCR has not been experimentally adressed. Our results show that knockout or knockdown of the *HMGN1* gene, either alone or in combination with the related *HMGN2* gene, does not impair TCR in at least two different human cell-lines. Obtaining identical results in osteosarcoma (U2OS) and retinal epithelial cells (RPE1), suggests that our findings are not cell-line specific.

Several functional assays were performed to monitor a functional role in TCR, including clonogenic survival assays after exposure to either Illudin S or UV light, which both trigger transcription-blocking DNA lesions, or recovery of RNA synthesis (RRS) assays, which measure the ability of cells to restart transcription following UV irradiation. While cells knockout for either the *XPA* and *CSB* genes, which are essential for TCR, displayed pronounced defects in all these assays, all the HMGN1-KO or HMGN1/2-dKO clones were indistinguishable from wild-type cells. Thus, our results strongly suggest that HMGN1 and HMGN2 are not required for human TCR.

### Human HMGN1 does not associate with stalled RNAPIIo or TCR proteins

Immunoprecipitation experiments do not support an association of HMGN1 with DNA damage-stalled RNAPIIo, or with either CSB or CSA in response to UV irradiation. These findings are in line with our functional analysis, and strongly suggest that HMGN1 is not part of the TCR complex. Under the same conditions, we could readily detect a strong UV-induced association between RNAPIIo, CSB, CSA and the TFIIH complex, arguing that our exerimental conditions would allow us to detect the association of HMGN1 if it would occur. Nonetheless, we could neither detect endogenous HMGN1 in CSB precipitates, nor could we detect TCR factors in HMGN1-GFP precipitates. Thus, our interaction experiments do not support an association of HMGN1 with the human TCR complex. Moreover, we show that the association of known TCR factors with DNA damage-stalled RNAPIIo is not affected by the combined loss of the *HMGN1* and *HMGN2* genes.

### Genetic differences between human and murine TCR

Human HMGN1 is a small (100 amino acid) protein with striking sequence conservation (83%) compared to mouse HMGN1 (96 amino acids; Supplementary Fig. 1A). In fact, the sequence conservation between human and mouse HMGN1 (83%) is far greater than that between human HMGN1 and HMGN2 (47%; Supplementary Fig. 1B,C). However, our findings suggest that human HMGN1 is not required for human TCR, while a previous study reported that UV-induced lesions in transcribed genes are repaired with decreased efficiency in HMGN1-deficient mouse cells^27^. Importantly, the UV-sensitive phenotype of these HMGN1-KO mouse cells could be rescued by re-expression of wild-type human HMGN1, but not by mutants that are either unable to bind to nucleosomes, or unable to unfold chromatin, suggesting that this phenotype is a specific effect of the loss of the *HMGN1* gene in mice^27^.

The strong evolutionary similarity (83%) between human and mouse HMGN1 suggest that it is unlikely that the function of HMGN1 between these species is not conserved due to changes at the protein level. However, there are fundamental differences between the organization of TCR in humans compared to mice that are much more likely to underlie these species-specific differences. In humans, the GGR sub-pathway of NER recognizes and removes UV-induced CPDs through the DDB2 damage-recognition factor^36–39^. Indeed, inherited mutations in DDB2 cause a predisposition to develop skin cancer^40^. In contrast, mice are largely deficient in the removal of CPDs by GGR owing to very low expression levels of DDB2^41^, and instead rely on TCR to remove CPDs from their genome during transcription. Consequently, TCR-deficient CSB−/− and CSA−/− mice develop skin cancer^42,43^ due to their inability to repair CPDs, which is not observed in human Cockayne syndrome (CS) patients^44^. Conversely, CS mice do not display strong neurological features^42,43^, which is a defining hallmark of CS in human patients^44^, further illustrating differences between mice and man when it comes to TCR deficiency.

We propose that human cells may not have a need for HMGN1-mediated chromatin modulation to remove CPDs during TCR, because these lesions are targeted by DDB2-mediated GGR. Indeed, several studies have revealed that DDB2 mediates higher-order chromatin unfolding at sites of UV-induced DNA damage^45,46^ similar to what has been proposed for HMGN1 in mouse cells^26^.

In conclusion, our findings strongly suggest that the role of murine HMGN1 in transcription-coupled DNA repair is not conserved in human cells.

## Experimental Procedures

### Cell lines

All human cells (listed in Supplementary Table 1) were cultured at 37 °C in an atmosphere of 5% CO2 in DMEM (Thermo Fisher Scientific) supplemented with penicillin/streptomycin (Sigma) and 10% Fetal bovine serum (FBS; Bodinco BV). Parental U2OS (WT) cells were a gift from Andreas Ladurner^47^. U2OS (FRT) cells containing the Flp-In^TM^/T-REx^TM^ system (Thermo Fisher Scientific), were a gift from Daniel Durocher^48^. All cell lines tested negative for mycoplasma contamination.

### Generation of knockout cell lines

To generate single knockouts, U2OS (FRT) cells were co-transfected with pLV-U6g-PPB encoding a guide RNA from the LUMC/Sigma-Aldrich sgRNA library (see Supplementary Table 2 for plasmids, Supplementary Table 3 for sgRNA sequences) targeting the *HMGN1*, *CSB* or *XPA* gene together with an expression vector encoding Cas9–2A-GFP (pX458; Addgene #48138) using lipofectamine 2000 (Invitrogen). Transfected cells were selected on puromycin (1 µg/mL) for 3 days, plated at low density after which individual clones were isolated. To generate HMGN1/HMGN2 double knockouts, U2OS (WT) or RPE1-hTERT cells were co-transfected with pLV-U6g-PPB sgHMGN1-2 and pX458 sgHMGN2 also encoding Cas9-2A-GFP. Transfected cells were FACS sorted on BFP/GFP, plated at low density after which individual clones were isolated. Isolated knockout clones were verified by western blot analysis and/or sanger sequencing. The absence of Cas9 integration/stable expression was confirmed by western blot analysis.

### Knockdown of HMGN1 or HMGN2

Cells were transfected twice with siRNAs at 0 and 36 hrs and were typically analyzed 60 hrs after the first transfection. All siRNA transfections (see Supplementary Table 4 for siRNA sequences) were performed with 40 siRNA duplexes using Lipofectamine RNAiMAX (Invitrogen) in OptiMEM without FBS.

### Generation of stable cell lines

Selected knockout clones of CSB and HMGN1 (see Supplementary Table 1) were subsequently used to stably express GFP-CSB, or HMGN1-GFP by co-transfection of pCDNA5/FRT/TO-Puro plasmid encoding these genes (5 µg), together with pOG44 plasmid encoding the Flp recombinase (0.5 µg). After selection on 1 µg/mL puromycin and 4 µg/mL blasticidin S, single clones were isolated and expanded. Clones were selected based on their near-endogenous expression level of GFP-tagged proteins compared to parental U2OS Flp-In/T-REx cells. Expression of these GFP-tagged proteins was induced by the addition of 2 µg/ml Doxycycline for 24 hrs.

### Plasmid constructs

To insert sgRNA sequences targeting HMGN2 into pX458, two oligonucleotides (see Supplementary Tables 3 and 5) were annealed in annealing buffer (100 mM NaCl, 50 mM HEPES; pH 7,4) by boiling for 5 min in water after which the mixture was allowed to cool down to room temperature. Annealed oligonucleotides were inserted into BbsI-digested pX458 plasmid. The Neomycin resistance gene in pcDNA5/FRT/TO-Neo (Addgene #41000) was replaced with a Puromycin resistance gene. Fragments spanning GFP-N1 (clontech) including the multiple cloning site were inserted into pcDNA5/FRT/TO-puro. The HMGN1 cDNA was inserted as an XhoI/BsrGI fragment into pcDNA5/FRT/TO-Puro-GFP-N1. All sequences were verified by sequencing.

### Clonogenic survival assays

Parental and knockout cell lines were trypsinized, seeded at low density and mock-treated or exposed to an increasing dose of UV light (0.5, 1, 2 J/m2 of UV-C 266 nm) or an increasing dose of Illudin S (Santa cruz; sc-391575) for 72 h (15, 30, 60, 100 pg/mL). On day 10, the cells were washed with 0.9% NaCl and stained with methylene blue. Colonies of more than 20 cells were scored. Survival experiments were conducted in triplicate and repeated at least three times.

### Immunoprecipitation for Co-IP

Cells were UV Irradiated (20 J/m2) or mock treated and harvested 1 h after UV. Chromatin-enriched fractions were prepared by incubating the cells for 20 min on ice in IP-150 buffer (50 mM Tris pH 7.5, 150 mM NaCl, 0.5% NP-40, 2 mM MgCl_2_ with protease inhibitor cocktail (Roche)), followed by centrifugation, and removal of the supernatant. For GFP-IPs, the chromatin-enriched cell pellets were subsequently lysed in IP-150 buffer supplemented with 500 U/mL Benzonase Nuclease (Novagen) for 1 h at 4 °C. For endogenous RNA pol II IPs, the chromatin-enriched cell pellets were lysed in IP-150 buffer supplemented with 500 U/mL Benzonase Nuclease (Novagen) and 2 µg RNAPII-S2 (ab5095, Abcam) for 1 h at 4 °C, followed by adding concentrated NaCl to increase the NaCl concentration to 300 mM and incubation of another 30 minutes at 4 °C. Protein complexes were pulled down by 1.5 h incubation with Protein A agarose beads (Millipore; endogenous RNA pol II IPs) or GFP-Trap®_A beads (Chromotek; GFP IPs). For subsequent analysis by western blotting, samples were prepared by boiling in Laemmli-SDS sample buffer.

### Western blot

Cells were spun down, washed with PBS, and boiled for 10 minutes in Laemmli buffer (40 mM Tris pH 6.8, 3.35% SDS, 16.5% glycerol, 0.0005% Bromophenol Blue and 0.05 M DTT). Proteins were separated on 4–12% Criterion XT Bis-Tris gels (Bio-Rad, #3450124) in NuPAGE MOPS running buffer (NP0001-02 Thermo Fisher Scientific), and blotted onto PVDF membranes (IPFL00010, EMD Millipore). Membranes were blocked with blocking buffer (Rockland, MB-070-003) for 2 h at RT, and probed with the indicated antibodies (listed in Supplementary Table 6). An Odyssey CLx system (LI-COR Biosciences) was used for detection.

### RNA recovery assay

30,000 cells were seeded on 12 mm glass coverslips in 24-wells plates in DMEM with 1% FBS. After 24 hours, cells were irradiated with UV-C at a dose of 6 J/m2 and incubated in conditioned medium for different time periods (0, 3 and 20 hours) to allow DNA repair and to restart RNA synthesis. Following incubation, nascent RNA was labelled by incubating the cells with 400 μM 5-ethynyluridine (5-EU; Jena Bioscience; CLK-N002-10,), which was then visualized with a click-iT mix consisting of 50 mM Tris buffer pH8, 60 μM Atto Azide (ATTO-TEC; 647N-101), 4 mM CuSO4•5H2O, 10 mM L-ascorbic acid (Sigma-Aldrich; A0278) and 1:1000 DAPI (ThermoFisher; D1306) for one hour. Cell were washed three times for 5 minutes with PBS, and mounted on microscope slides (Thermo Scientific) using Aqua Polymount (Polysciences, Inc. #18606). All RRS experiments were conducted in triplicate and repeated at least three times.

### Microscopic analysis of fixed cells

Images of fixed samples were acquired on a Zeiss AxioImager M2 or D2 widefield fluorescence microscope equipped with 63x PLAN APO (1.4 NA) oil-immersion objectives (Zeiss) and an HXP 120 metal-halide lamp used for excitation. Fluorescent probes were detected using the following filters for DAPI (excitation filter: 350/50 nm, dichroic mirror: 400 nm, emission filter: 460/50 nm) and Alexa 555 (excitation filter: 545/25 nm, dichroic mirror: 565 nm, emission filter: 605/70 nm). Images were recorded using ZEN 2012 software and analyzed in Image J.

### UV-C laser microscopy

Cells were grown on 18 mm Quartz coverslips and placed in a Chamlide CMB magnetic chamber in which growth medium was replaced by CO_2_-independent Leibovitz’s L15 medium. Laser tracks were made by a diode pumped solid state 266 nm Yttrium Aluminum Garnet laser (Average power 5 mW, repetition rate up to 10 kHz, pulse length 1 ns) in a UGA-42-Caliburn/2 L Spot Illumination system (Rapp OptoElectronic) with laser power set to 20%. This was combined with live-cell imaging in an environmental chamber set to 37 °C on an all-quartz widefield fluorescence Zeiss Axio Observer 7 microscope, using a 100 × 1.2 NA glycerol objective. The laser system is coupled to the microscope via a triggerbox and a neutral density (ND-1) filter is installed to block 90% of the laser light. A HXP 120 V metal-halide lamp was used for excitation.

### CPD staining

Cells were grown on a 18 mm glass coverslip and globally UV irradiated (20 J/m2). After irradiation, the cells were fixed with 3.7% PFA at indicated time points. Cells were permeabilized for 20 min in PBS with 0.5% Triton-X100 and subsequently blocked with 100 mM Glycine. DNA was denatured with 0.5% NaOH for 5 min, followed by blocking with 10% BSA (Thermo Fisher) for 15 min. Next, the cells were incubated first with the antibody against CPDs (see Supplementary Table 5) for 2 h then the secondary antibody anti-mouse Alexa 555 for 1 h and last with DAPI for 5 min. Lastly the cells were mounted in mounted in Polymount (Brunschwig).

### Statistical analysis

The statistical analysis was performed using GraphPad Prism 8 software (GraphPad Soſtware). Results were compared to WT cells and analyzed using one-way ANOVA followed by Dunnet’s multiple comparisons test. Values of p < 0.05 were considered statistically significant.

## Supplementary information

Supplementary Information

## Data Availability

The datasets generated and analysed in the current study are available from the corresponding author upon reasonable request.
