# Supplementary material for: Human HMGN1 and HMGN2 are not required for transcription-coupled DNA repair
Source: Sci Rep. 2020 Mar 9;10:4332. doi: 10.1038/s41598-020-61243-4 (PMC7062826; doi:10.1038/s41598-020-61243-4)

Human HMGN1 and HMGN2 are not required for transcription-coupled DNA repair

Katja Apelt[1], Iris Zoutendijk[1], Dennis Y. Gout[1], Annelotte P. Wondergem[1], Diana van den Heuvel[1], and Martijn S. Luijsterburg[1,*]

[1] Department of Human Genetics, Leiden University Medical Center, Einthovenweg 20, 2333 ZC, Leiden, The Netherlands

Running title: HMGN1 is not involved in TCR

* Corresponding author: Martijn Luijsterburg (m.luijsterburg@lumc.nl)

Supplementary Figure 1

a

```
                   NLS            NBD                                  NLS
HMGN1_HUMAN     MPKRKVSSAEGAAKEEPKRRSARLSAKP-PAKVEAKPKKAAAKDKSSDKKVQTKGKRGAK     59
HMGN1_MOUSE     MPKRKVS-ADGAAKAEPKRRSARLSAKPAPAKVDAKPKKAAGKDKASDKKVQIKGKRGAK     59
                ******* *:**** ************* ****:*******.***:****** *******

                                CHUD
HMGN1_HUMAN     GKQAEVANQETKEDLPAENGETKTEESPASDEAGEKEAKSD 100
HMGN1_MOUSE     GKQADVADQQTT-ELPAENGETENQS-PASEE--EKEAKSD 96
                ****:**:*:*. :********:.:. ***:*  *******
```

b

```
                                       NBD                            NLS
                  NLS
HMGN1_HUMAN     MPKRKVSS----AEGAAKEEPKRRSARLSAKPPA-KVEAKPKKAAAKDKSSDKKVQTKGK     55
HMGN2_HUMAN     MPKRKAEGDAKGDKAKVKDEPQRRSARLSAKPAPPKPEPKPKKAPAKKGE----KVPKGK     56
                *****...     :. .*:**:**********   * * ***** **. .       ***

                NLS           CHUD
HMGN1_HUMAN     RGAKGKQAEVANQETKEDLPAENGETKTEESPASDEAGEKEAKSD       100
HMGN2_HUMAN     KGKA-------DAGKEGNNPAENGDAKTDQAQKAEGAGDAK----       90
                :*         :  .: : *****::**:::  :: **: :
```

c

```
                   NLS                   NBD                             NLS
HMGN1_HUMAN     MPKRKVSS------AEGAAKEEPKRRSARLSAKPP-AKVEAKPKKAAAKDKSSDKKVQTK     53
HMGN2_HUMAN     MPKRKAEGDAKG--DKAKVKDEPQRRSARLSAKPAPPKPEPKPKKAPAKK--G-----EK     51
HMGN3_HUMAN     MPKRKSPENTEGKDGSKVTKQEPTRRSARLSAKPAPPKPEPKPRKTSAKKEPG-----AK     55
HMGN4_HUMAN     MPKRKAKGDAKG--DKAKVKDEPQRRSARLSAKPAPPKPEPRPKKASAKK--G-----EK     51
                *****          .  .*:** **********   * * :*:*: **.  .      *

                  NLS                CHUD
HMGN1_HUMAN     GKRGAKGKQAEVANQETKEDLPAENGETKTEESPASDEAGEKEAKSD    100
HMGN2_HUMAN     VPKGKKG----KADAGKEGNNPAENGDAKTDQAQKAEGAGDAK----    90
HMGN3_HUMAN     ISRGAKGKKEEKQEAGKEGTAPSENGETKAEEAQKTESVDNEGE---    99
HMGN4_HUMAN     LPKGRKG----KADAGKDGNNPAKNRDASTLQSQKAEGTGDAK----    90
                  :* **      :  ..   *::* ::.: ::  :: ..:
```

Supplementary Figure 1. Alignment of HMGN proteins. (a) Sequence alignment of human and mouse HMGN1 proteins. (b) Sequence alignment of human HMGN1 and HMGN2 proteins. (C) Sequence alignment of human HMGN1, HMGN2, HMGN3, and HMGN4 proteins. All sequences were aligned with ClustalW. NLS = nuclear localization signal, NBD = nucleosome-binding domain, CHUD = chromatin-unfolding domain. Uncropped Western blot data is shown in the Supplementary Information file.

Supplementary Figure 2

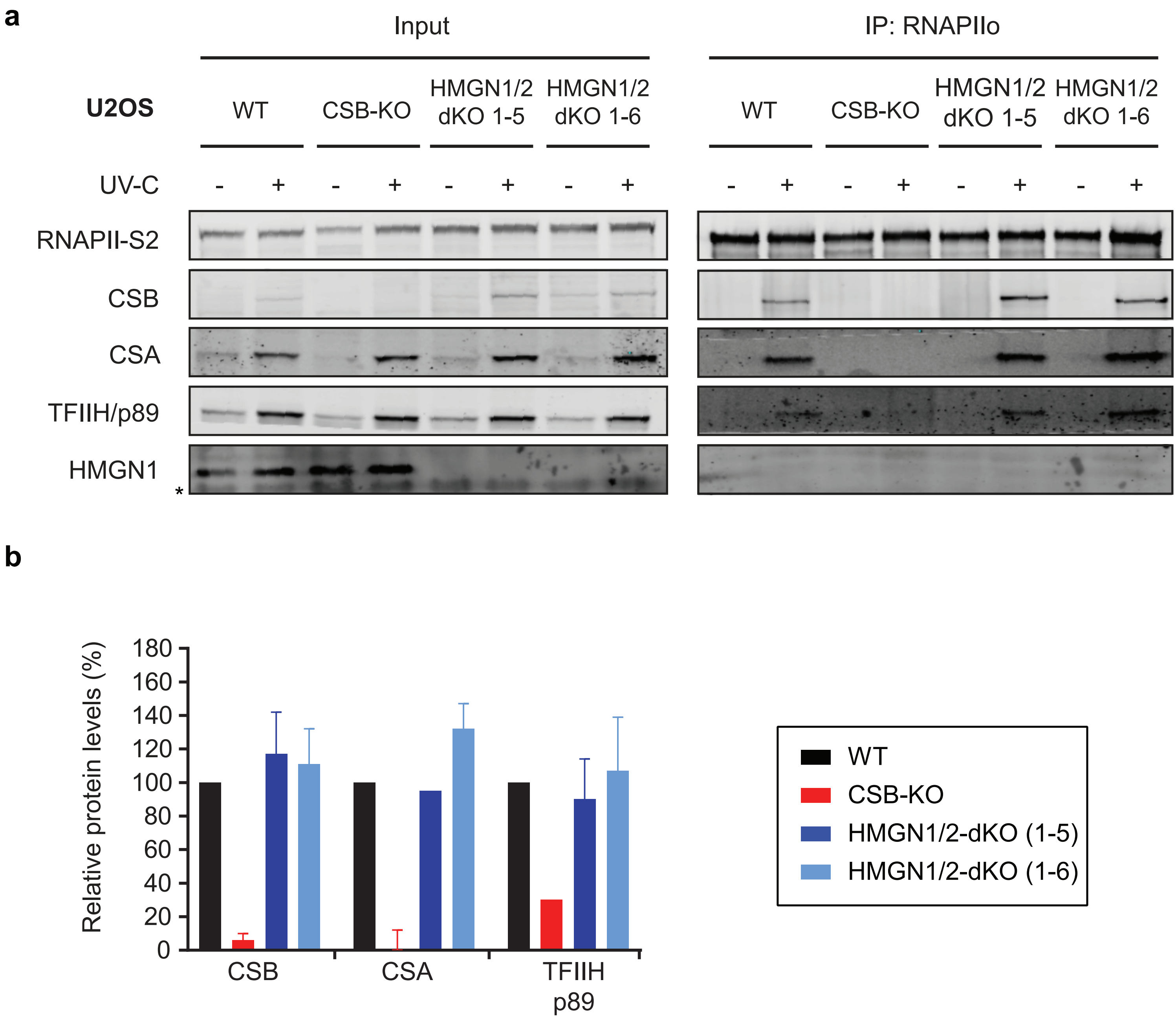

Supplementary Figure 2. (a) Repeat of Co-IP of endogenous RNAPIIo in unirradiated or UV-irradiated U2OS WT, CSB-KO and HMGN1/HMGN2-dKO cells and (b) Quantification of relative protein levels. Data represent mean ± SEM of two independent experiments. Uncropped Western blot data is shown in the Supplementary Information file.

Supplementary Figure 3

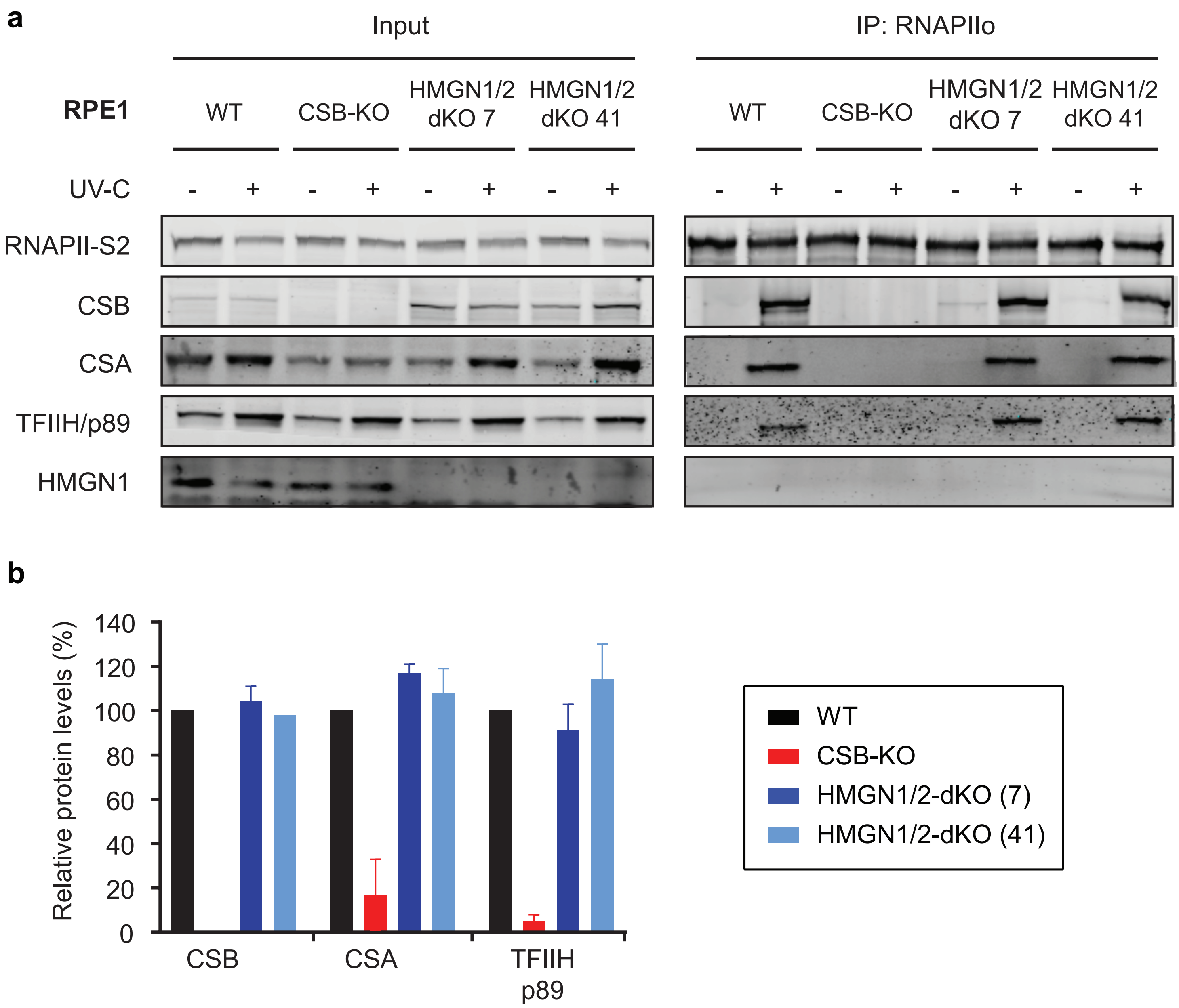

Supplementary Figure 3. (a) Repeat of Co-IP of endogenous RNAPIIo in unirradiated or UV-irradiated RPE1 WT, CSB-KO and HMGN1/HMGN2-dKO cells and (b) Quantification of relative protein levels. Data represent mean ± SEM of two independent experiments. Uncropped Western blot data is shown in the Supplementary Information file.

Table 1: Cell lines

**Cell lines**	**Origin**
RPE1-hTERT	This study
RPE1-hTERT CSB-KO (15)	This study
RPE1-hTERT HMGN1/HMGN2-dKO (41)	This study
RPE1-hTERT HMGN1/HMGN2-dKO (7)	This study
RPE1-hTERT XPC-KO (5)	This study
U2OS (FRT)	[48]
U2OS (FRT) CSB-KO (1-12)	This study
U2OS (FRT) CSB-KO (1-12) + GFP-CSB (3)	This study
U2OS (FRT) HMGN1-KO (1-9)	This study
U2OS (FRT) HMGN1-KO (2-11)	This study
U2OS (FRT) HMGN1-KO (2-4)	This study
U2OS (FRT) HMGN1-KO (2-4) + HMGN1-GFP (9)	This study
U2OS (FRT) XPA-KO (2-8)	This study
U2OS (WT)	[47]
U2OS (WT) EGFP-RPB1	[34]
U2OS (WT) HMGN1/HMGN2-dKO (1-5)	This study
U2OS (WT) HMGN1/HMGN2-dKO (1-6)	This study

Table 2: Plasmids

**Plasmids**	**Origin**
pcDNA5/FRT/TO-Neo	Addgene #41000
pcDNA5/FRT/TO-Puro	This study
pcDNA5/FRT/TO-Puro-GFP-N1	This study
pcDNA5/FRT/TO-Puro-HMGN1-GFP	This study
pEGFP-N1	Clontech
pHMGN1-EGFP	[27]
pLV-U6g-PPB	LUMC/Sigma-Aldrich sgRNA library
pLV-U6g-PPB sgCSB	This study
pLV-U6g-PPB sgHMGN1-1	This study
pLV-U6g-PPB sgHMGN1-2	This study
pLV-U6g-PPB sgXPA	This study
pLV-U6g-PPB sgXPC	This study
pOG44	Thermo Fisher
pX458	Addgene #48138
pX458 sgHMGN2-1	This study
pX458 sgHMGN2-2	This study

Table 3: Sequences of sgRNAs

**sgRNAs**	**Sequence**
sgCSB	CCAGACTTCAAGTCACAAAGTTG
sgHMGN1-1	GTAAGTCTTCTTTAGTTTCTTGG
sgHMGN1-2	CCTCAGTCTTCGTTTCCCCGTTT
sgHMGN2-1	TGCTAAGGGAGATAAAGCAA
sgHMGN2-2	GGGAGATAAAGCAAAGGTGA
sgXPA	CCTGTGTCAATTATCTTTGGGGC
sgXPC	CCGAAGATATGTCTCAAACTCCA
	TGGGGGTTTCTCATCTTCAAAGG

Table 4: Sequences of siRNAs

**siRNAs**	**Sequence**
siLuc	CGTACGCGGAATACTTCGA
siXPA	CAGAGATGCTGATGATAAA
siHMGN1-1	GGATAAATCTTCAGACAAA
siHMGN2-1	AAGCAAAGGTGAAGGACGA
siHMGN1-2	TGATGAAGCAGGAGAGAAA

Table 5: Primers

**Primers**	
HMGN2-1 (sgRNA)	CACCGTGCTAAGGGAGATAAAGCAA
	AAACTTGCTTTATCTCCCTTAGCAC
HMGN2-2 (sgRNA)	CACCGGGGAGATAAAGCAAAGGTGA
	AAACTCACCTTTGCTTTATCTCCCC

Table 6: Antibodies

**Antibodies**	**Host**		**Clone**	**WB**	
Cas9	Mouse	Cell Signaling technology, #14697	7A9 and 3A3	1/5000	aML#031
CPD	Mouse	Cosmo Bio CAC-NM-DND-001		1:1500	aML#020
CSA/ERCC8	Mouse	Santa Cruz, sc-376981	D2	1/500	aML#025
CSA/ERCC8	Rabbit	Abcam, 137033	EPR9237	1/750	aML#028
CSB/ERCC6	Goat	Santa Cruz, SC-10459	E-18	1/1000	aML#039
GFP	Mouse	Roche, #11814460001	7.1 and 13.1	1/1000	aML#011
GFP	Rabbit	Abcam, ab290		1/1000	aML#044
HMGN1	Rabbit	Cell Signaling 5692	#5692	1:1000	aML#058
HMGN2	Rabbit	Cell Signaling 9437		1:1000	aML#065
Mouse IgG (H+L) CF770	Goat	Biotium, VWR #20077		1/10000	aML#009
p89/TFIIH	Mouse	Millipore, MABE1123	15TF2-1B3	1/2000	aML#101
rabbit IgG (H+L) CF680	Goat	Biotium, VWR #20067		1/10000	aML#010
RNAPIIo	Rabbit	Abcam, ab5095		1/1000	aML#024
Tubulin	Mouse	Sigma, T6199	DM1A	1/1000	aML#008
XPA	Rabbit	kindly provided by Rick Wood	CJ1	1/10000	aML#079
XPC	Rabbit	Novus Biologicals NB100-58801		1:1000	aML#077

Uncropped WB file from figure 1A

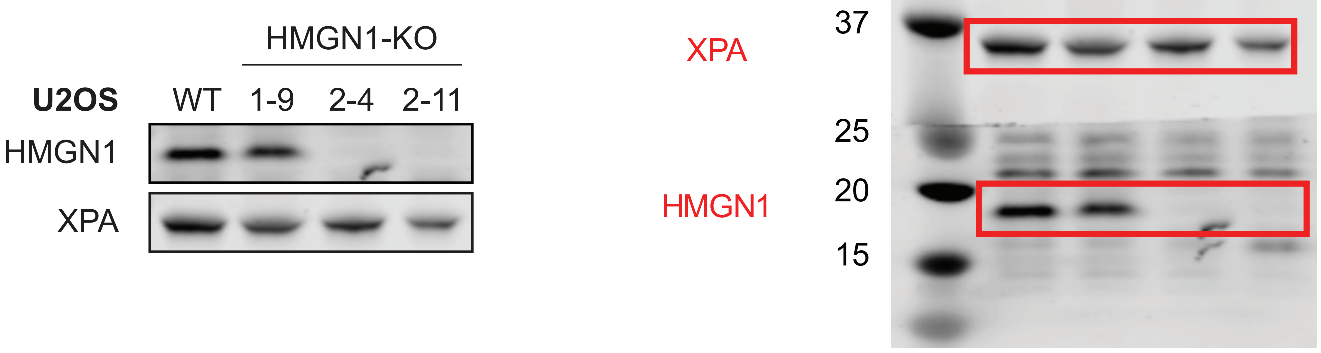

Uncropped WB file from figure 1B

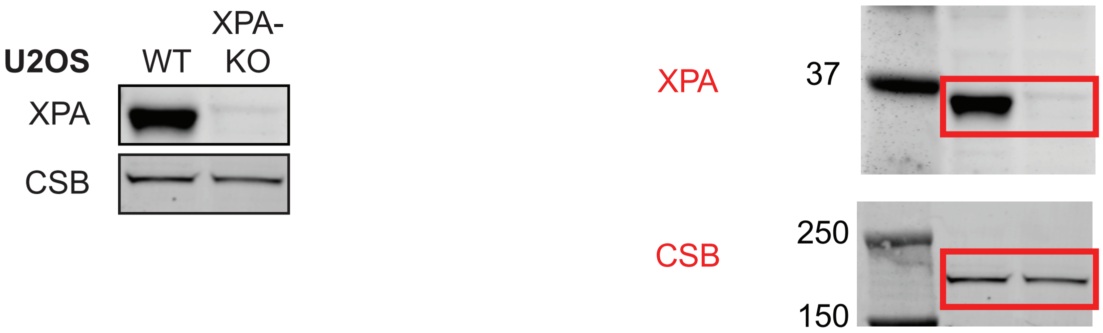

Uncropped WB file from figure 2A

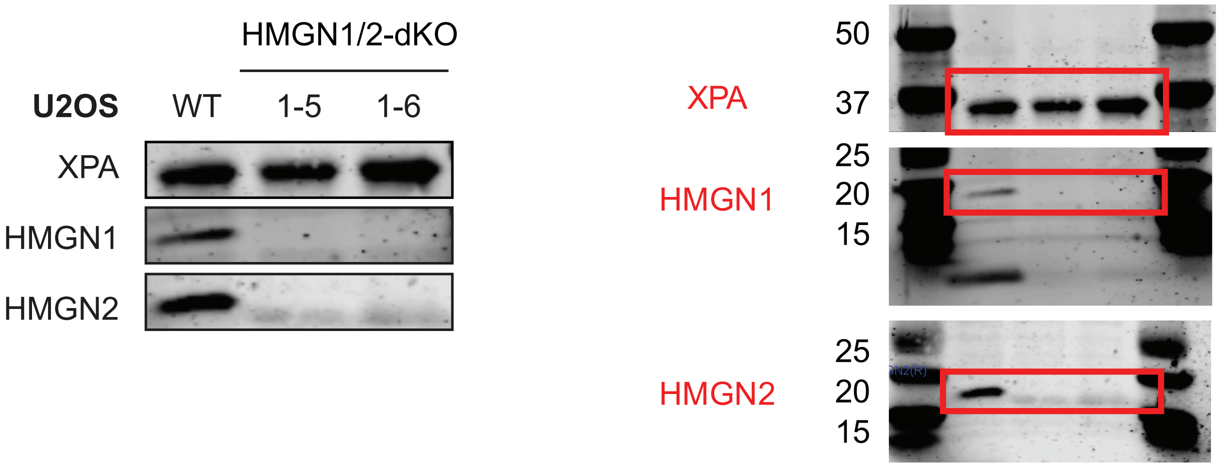

Uncropped WB file from figure 2B

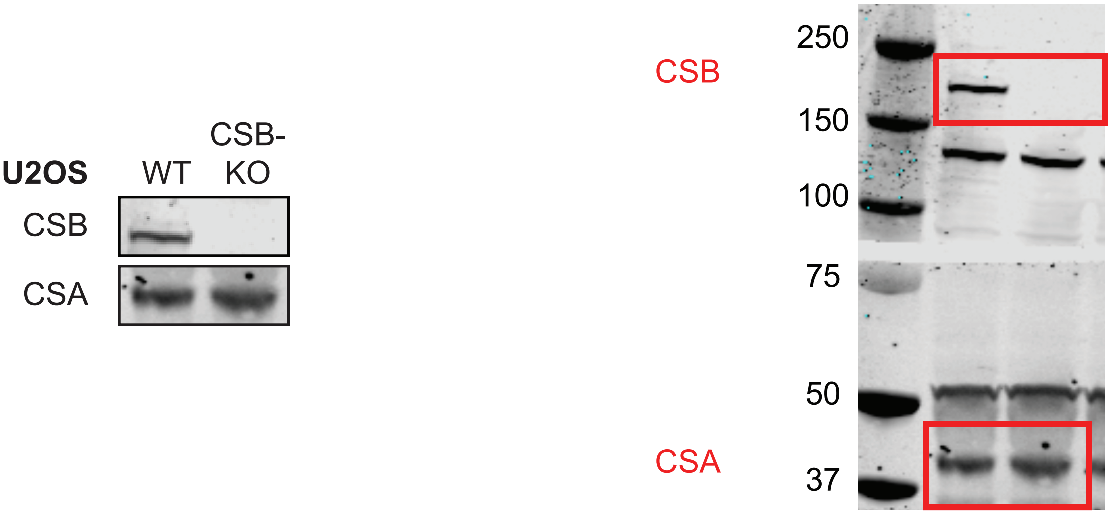

Uncropped WB file from figure 3A

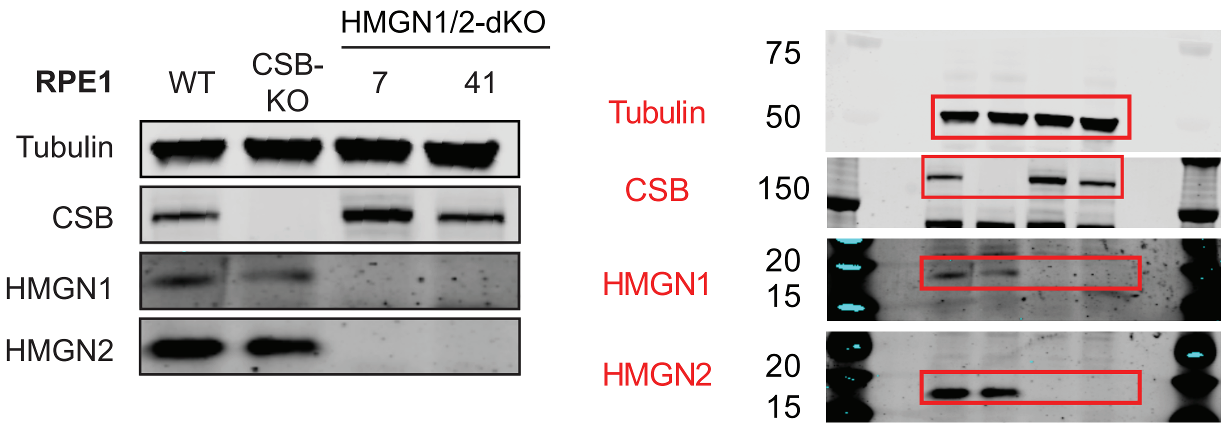

Uncropped WB file from figure 4A

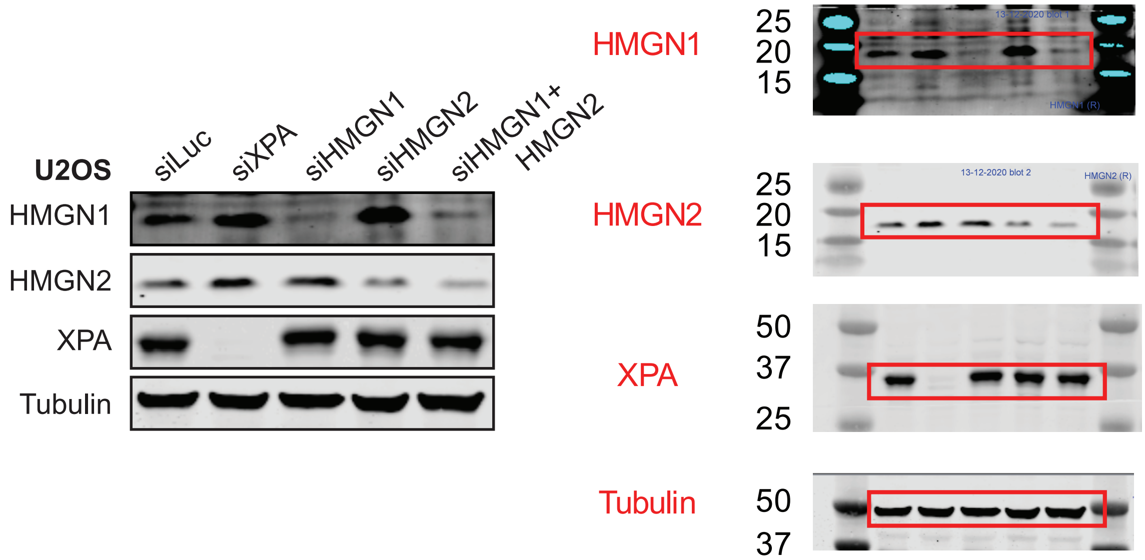

Uncropped WB file from figure 6B

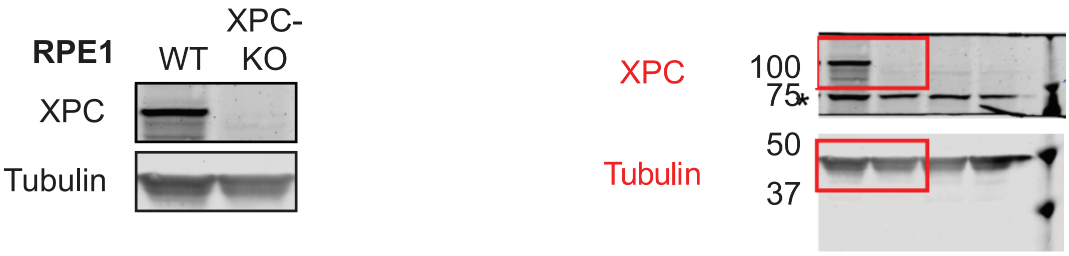

Uncropped WB file from figure 7A

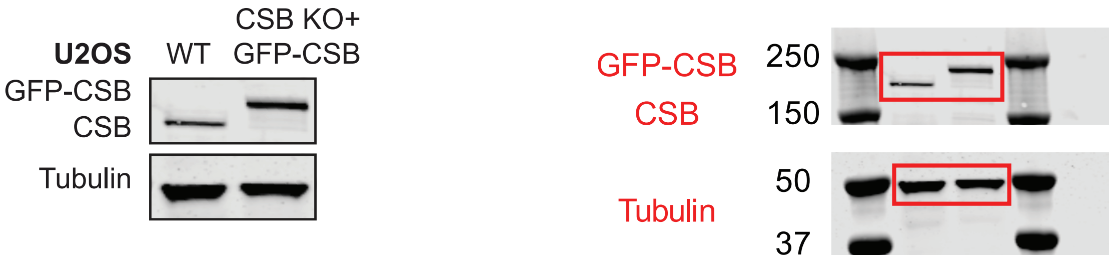

Uncropped WB file from figure 7B

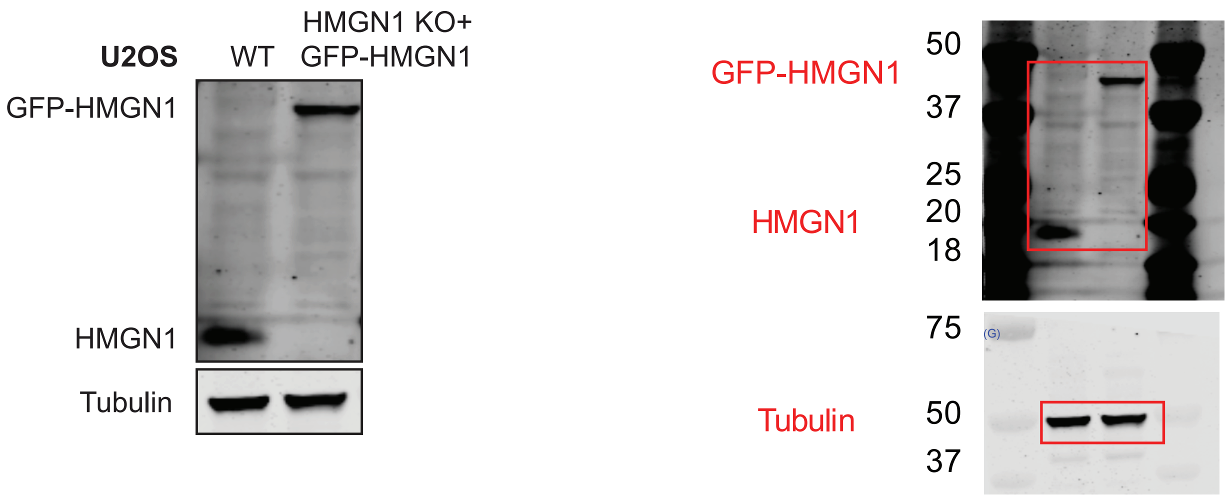

Uncropped WB file from figure 7D

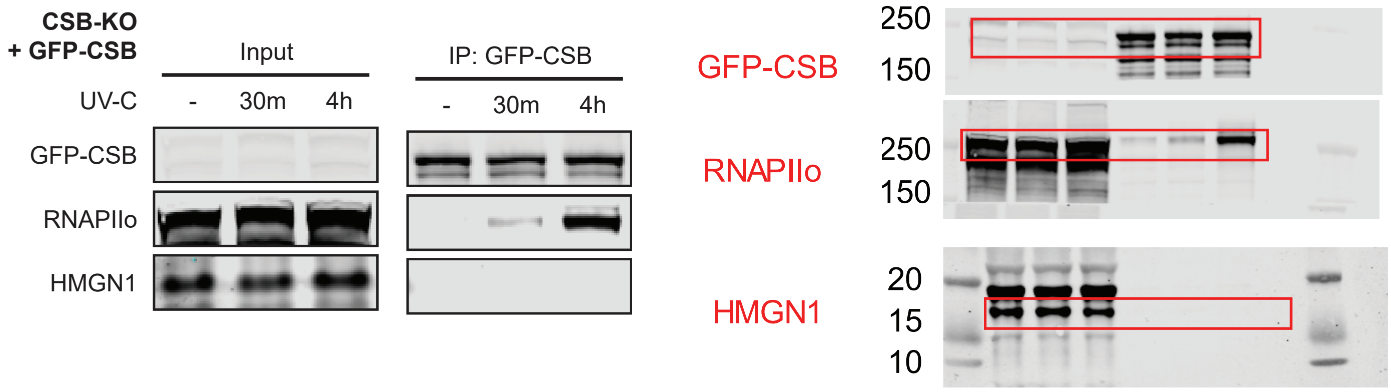

Uncropped WB file from figure 7E

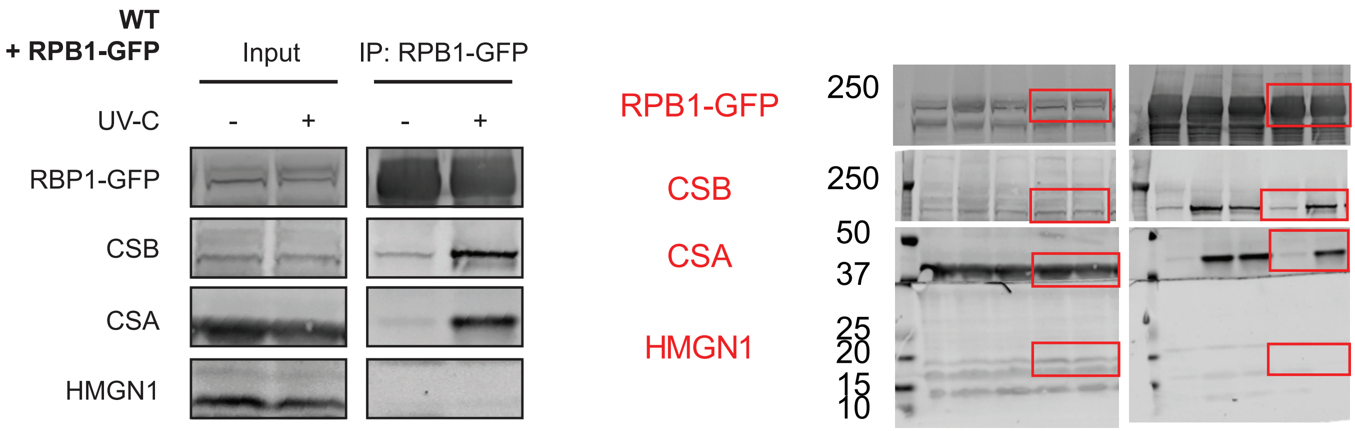

Uncropped WB file from figure 7F

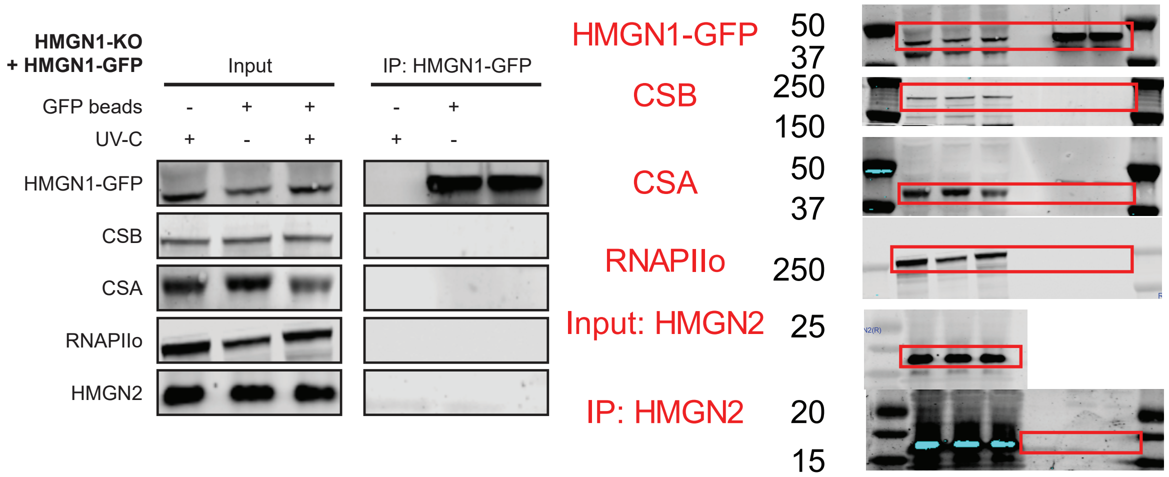

Uncropped WB file from figure 8A

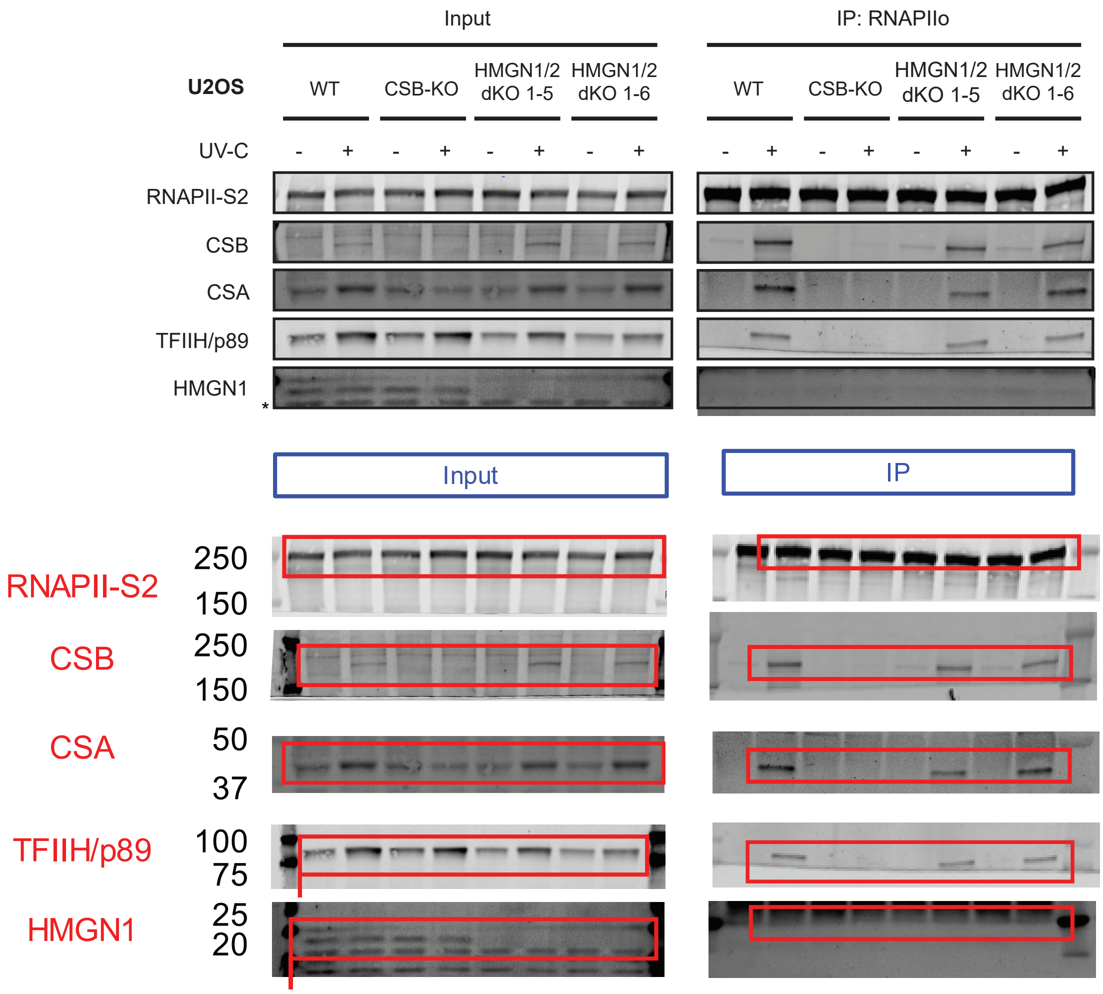

Uncropped WB file from figure 8B

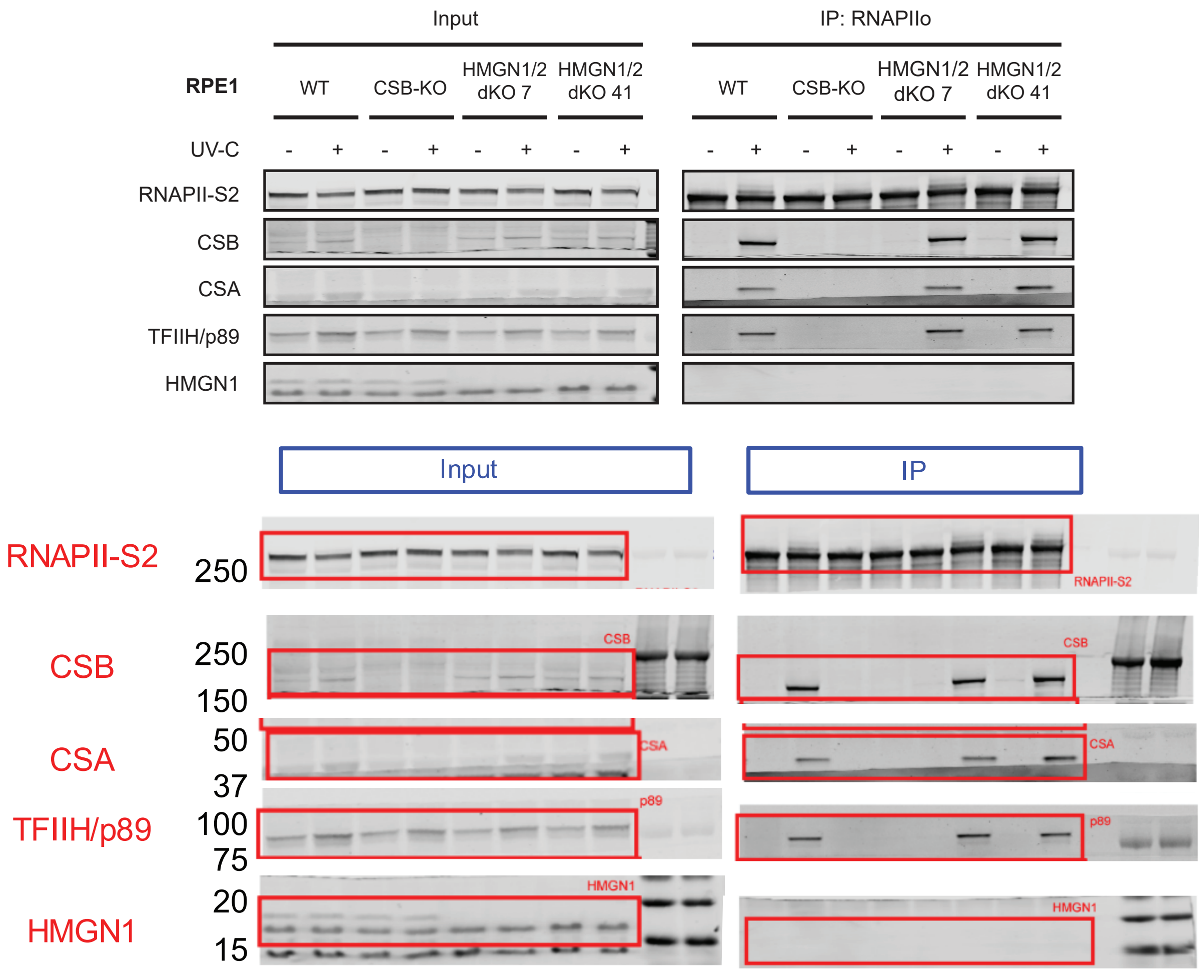

Uncropped WB file from supplementary figure 2A

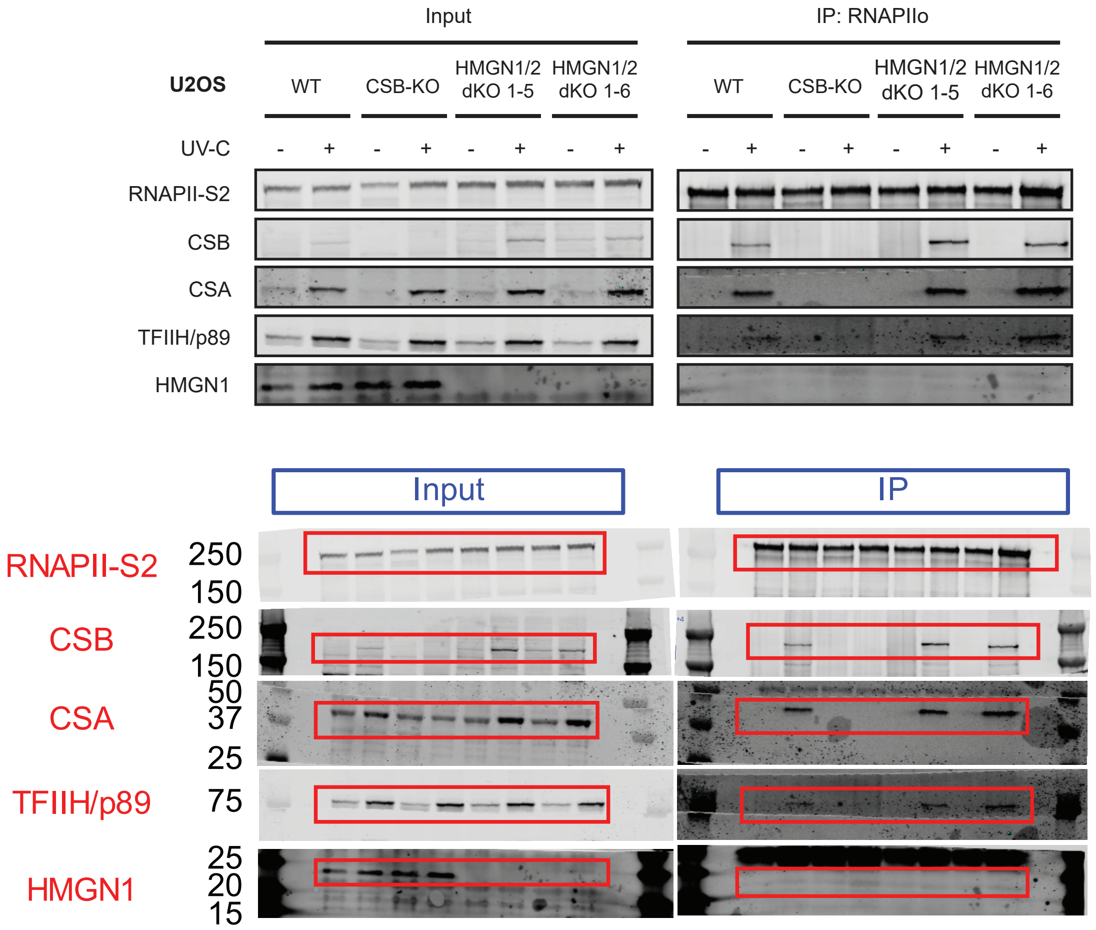

Uncropped WB file supplementary figure 3A

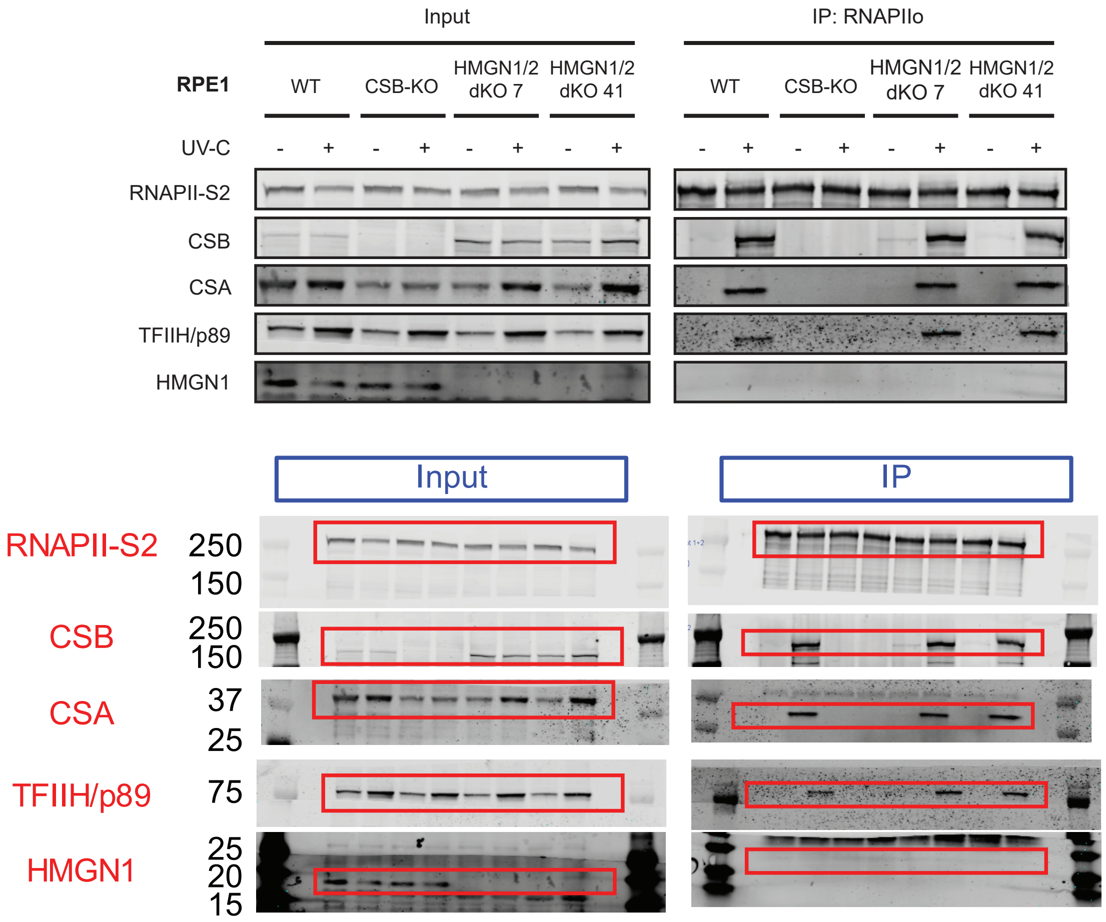

Supplement: Supplementary file 1 — Supplementary Information [file 41598_2020_61243_MOESM1_ESM.pdf]
